# Toughening of Poly(hydroxyurethane)–Epoxy Hybrid Networks by High-Aspect-Ratio Cellulose Nanocrystals

**DOI:** 10.1021/acs.biomac.6c00531

**Published:** 2026-07-14

**Authors:** Pavithra M. Wijeratne, Li Zha, Connie Ocando, Lars A. Berglund, Jean-Marie Raquez, Qi Zhou

**Affiliations:** † Division of Glycoscience, Department of Chemistry, School of Engineering Sciences in Chemistry, Biotechnology and Health, KTH Royal Institute of Technology, 7655 AlbaNova University Centre, Stockholm 106 91, Sweden; ‡ Laboratory of Polymeric and Composite Materials, Department of Chemistry, Faculty of Science, 54521 University of Mons, Mons 7000, Belgium; § Department of Fiber and Polymer Technology, KTH Royal Institute of Technology, Stockholm 100 44, Sweden

## Abstract

Nonisocyanate polyurethanes are sustainable alternatives to conventional polyurethanes but often exhibit limited mechanical performance. Here, poly­(hydroxyurethane) (PHU)–epoxy hybrid networks were prepared to tune matrix stiffness through covalent cross-linking and reinforced with wood- and tunicate-derived cellulose nanocrystals (CNCs). Increasing epoxy content enhanced Young’s modulus and tensile strength while reducing strain at break. In contrast, incorporation of CNCs into an intermediate PHU–epoxy matrix produced substantial mechanical enhancement at low loadings. Tunicate-derived CNCs, characterized by higher aspect ratios, provided greater reinforcement than wood-derived CNCs and achieved a maximum toughness of ∼27 MJ m^–3^ at 2 wt %, exceeding both wood-derived CNC nanocomposites (≤17 MJ m^–3^) and neat PHU–epoxy hybrids (≤16.5 MJ m^–3^). Scanning electron microscopy confirmed homogeneous CNC dispersion, while Fourier transform infrared spectroscopy indicated extensive hydrogen-bonding interactions. These results demonstrate that epoxy hybridization and high-aspect-ratio CNC reinforcement provide complementary routes to enhance the mechanical performance of biobased PHU materials.

## Graphical abstract

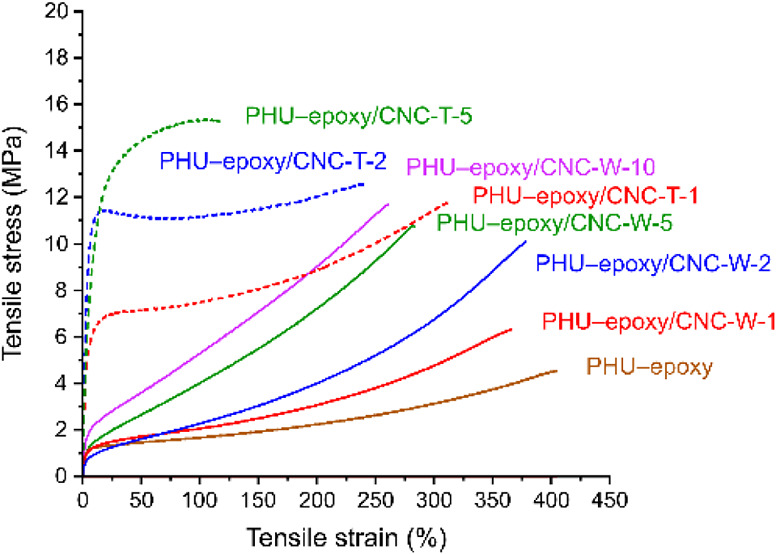

## 1 Introduction

Polyurethane (PU) is widely used in foams, coatings, elastomers, and adhesives and is conventionally synthesized by reacting isocyanates with polyols. However, isocyanates present serious health hazards, including asthma and dermatitis, and are produced from phosgene, a highly toxic gas associated with significant environmental concerns.^1,2^ These issues have driven increasing interest in nonisocyanate polyurethanes (NIPUs), in which aminolysis of cyclic carbonates has emerged as one of the most promising synthetic routes. This method avoids the use of isocyanates and yields poly­(hydroxyurethane)­s (PHUs) bearing primary and secondary hydroxyl groups along the backbone, which enable further interactions through hydrogen bonding. Despite these advantages, PHUs generally exhibit inferior mechanical properties compared with conventional PUs and suffer from drawbacks such as slow reaction kinetics, elevated reaction temperatures, long reaction times, low conversion, and undesired side reactions.^3–5^

Hybrid materials have been investigated as a strategy to improve PHU performance. Hybrid PHUs combine PHUs with other polymers or oligomers, such as acrylics, siloxanes, natural rubber, and lignin or tannin. Although promising, many of these hybrids encounter phase-compatibilization challenges.^2^ In contrast, PHU–epoxy hybrid polymers have demonstrated good compatibility and improved mechanical properties, partly attributed to increased network formation through cross-linking.^2,6–10^ Cornille et al. reported the synthesis of PHU prepolymers functionalized with primary amines that further reacted with epoxy to generate new classes of PHUs, which exhibited superior thermal performance compared to conventional PUs and PHUs.^8^ Figovsky et al. employed a two-step process using bisphenol resins and carbonated soybean oil to produce hybrid PHUs that combined the chemical resistance of epoxy resins with enhanced mechanical properties.^11^ More recently, PHU–epoxy hybrid networks have also been explored as covalent adaptable materials and recyclable composites, where epoxy incorporation improved dynamicity, processing, and thermomechanical performance.^12,13^ Hybrid PHU–epoxy systems have further been investigated for sustainable natural fiber composites and resin transfer molding applications, highlighting the potential of PHU-based hybrid matrices for structural composite materials.^14,15^

Nanocomposites based on PHUs reinforced with nanoscale fillers have also attracted growing attention for property enhancement. Fillers such as silica nanoparticles,^16–18^ carbon nanotubes,^19^ and gibbsite nanoplatelets^20^ have been incorporated into PHUs to improve mechanical performance. More recently, biomass-derived nanofillers including cellulose nanocrystals (CNCs) and chitin nanocrystals have also been explored in PHU systems.^21–25^ In our previous work, segmented thermoplastic PHU nanocomposites reinforced with CNCs and partially deacetylated chitin nanocrystals demonstrated that covalent compatibilization of chitin nanocrystals produced substantially greater reinforcement than noncovalent CNC interactions.^22^ In addition, CNCs and surface hydrophobically functionalized CNCs have recently been employed as stabilizers and reinforcing nanofillers in waterborne PHU latexes and adhesive systems.^23–25^ Among biomass-derived nanofillers, CNCs produced via controlled acid hydrolysis of cellulose fibers, are of particular interest due to their renewability, biodegradability, high elastic modulus (100–150 GPa), low density, and large specific surface area, making them promising candidates for reinforcing polymer matrices.^26^ CNCs derived from different biological sources exhibit distinct aspect ratios, which can influence stress transfer and reinforcement behavior. Moreover, the abundant hydroxyl groups in PHUs enable strong interfacial interactions with CNC surfaces through hydrogen bonding.

Despite these recent advances, the comparative roles of epoxy-driven hybridization and CNC-based reinforcement in PHU systems remain insufficiently understood. In particular, the influence of CNC aspect ratio on the balance between stiffness, strength, ductility, and toughness in PHU–epoxy hybrid networks have not been systematically investigated. In this work, we fabricated PHU–epoxy/CNC nanocomposites by curing PHU with epoxy in the presence of CNCs. The formation of a CNC network-like structure within the PHU–epoxy matrix was expected to promote strong interfacial adhesion and improve mechanical performance. CNCs derived from wood and tunicates were selected as reinforcing fillers, offering distinct aspect ratios due to their differences in fibril length. Tunicate-derived CNCs exhibit significantly higher aspect ratios than wood-derived CNCs, enabling investigation of aspect ratio effects on the thermomechanical properties of PHU nanocomposites. While epoxy incorporation into PHU increases network rigidity and strength through additional cross-linking, reinforcement with high-aspect ratio CNCs may enhance load transfer via network formation and hydrogen bonding. However, the relative contributions and trade-offs between matrix hybridization with epoxy and nanofiller reinforcement with CNCs remain insufficiently explored in PHU systems. Here, we compare how epoxy content and CNC type and loading influence stiffness, strength, ductility, and toughness. By analyzing PHU–epoxy hybrids alongside PHU–epoxy/CNC nanocomposites, we identify conditions under which matrix design or nanofiller reinforcement more strongly influences overall performance.

## 2 Experimental Section

### 2.1 Materials

Butanediol bis­(cyclocarbonate) (BCC, carbonate content of 6.8 mequiv g^–1^ was purchased from Specific Polymers. *m*-Xylylenediamine (*m*-XDA, Sigma-Aldrich X1202), bisphenol A diglycidyl ether (BADGE, Sigma-Aldrich D3415), 1,8-diazabicyclo[5.4.0]­undec-7-ene (DBU, Sigma-Aldrich 803282), anhydrous dimethylformamide (DMF), sodium chloride (NaCl), sodium chlorite (NaClO_2_), sodium hydroxide (NaOH), and deuterated chloroform (CDCl_3_) were purchased from Merck KGaA, Germany. All chemicals were used as received unless otherwise noted. Wood-derived CNCs (CNC-W, 0.85 wt % sulfur on dry CNC sodium form) were purchased from University of Maine, USA. The CNC-W suspension was dialyzed against deionized water and treated with a mixed-bed ion-exchange resin (Dowex Marathon MR-3), followed by filtration through Whatman 541 filter paper. *Ciona intestinalis* was provided by the Station Biologique de Roscoff (SBR), France, through the support from ASSEMBLE – Association of European Marine Biological Laboratories.

### 2.2 Preparation of Tunicate-Derived CNCs (CNC-T)

Cellulose was extracted from *Ciona intestinalis* by an initial alkaline treatment using sodium hydroxide to remove proteins, lipids, and other noncellulosic components, followed by three successive bleaching steps to eliminate residual pigments. The purified tunicate cellulose was mechanically disintegrated in water using a kitchen blender and subsequently freeze-dried. Sulfuric acid hydrolysis was then performed to prepare tunicate CNCs (CNC-T), following previously reported protocols.^27,28^ Briefly, the dried cellulose was mixed with sulfuric acid and hydrolyzed at 65 °C under constant stirring for 30 min. The resulting suspension was repeatedly rinsed with deionized water until neutral pH was reached, followed by mechanical disintegration through ultrasonication (Branson SFX550 Sonifier) until a stable, turbid supernatant was obtained. No sedimentation or flocculation was observed, which is consistent with the presence of surface sulfate ester groups introduced during acid hydrolysis. Both CNC-T and CNC-W samples were freeze-dried from a dilute aqueous suspension (0.05 wt %) and redispersed in DMF by ultrasonication (25% output, 2 min) without the use of additives or further chemical modification.

### 2.3 Synthesis of PHU–Epoxy Hybrid Polymer

The PHU prepolymer was synthesized under bulk conditions by reacting BCC with excess *m*-XDA in a sealed vial at 80 °C for 24 h, using DBU (5 mol % relative to cyclocarbonate groups) as the catalyst (Scheme 1). After prepolymer formation, BADGE dissolved in DMF was added to obtain a total solid content of 34 wt %. The mixture was stirred at 50 °C for 2 h and subsequently cast into a Teflon mold. Curing was carried out in a vacuum oven at 80 °C for 24 h, followed by postcuring at 120 °C for 4 h. The molar ratio of BCC:*m*-XDA:BADGE was varied from 1:1.1:0.2 to 1:1.5:1.0. The resulting PHU–epoxy hybrid films were transparent.

**Scheme 1:**
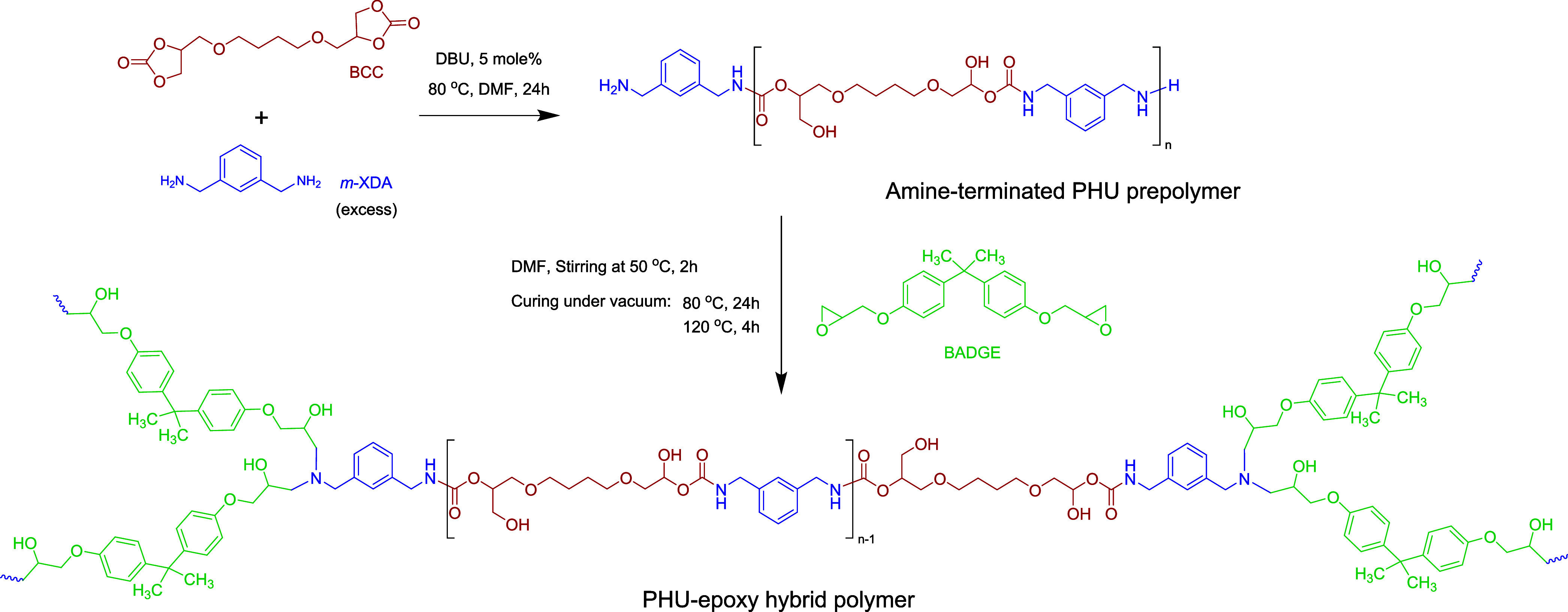
Synthetic Route to the PHU–Epoxy Hybrid Polymer[Fn sch1-fn1]

i DBU-catalyzed polyaddition of BCC with excess *m*-XDA produces an amine-terminated PHU prepolymer via aminolysis of the cyclic carbonate groups. Subsequent curing with BADGE yields a cross-linked PHU—Epoxy hybrid network.

### 2.4 Synthesis of PHU–Epoxy/CNCs Nanocomposites

The preparation of the nanocomposite films followed the synthesis protocol of PHU–epoxy hybrid polymer. The freeze-dried CNCs were redispersed in DMF together with BADGE. This mixture was then incorporated into the BCC/*m*-XDA prepolymer and stirred at 50 °C for 2 h, yielding a homogeneous polymer dispersion with a total solids content of 34 wt %. The selected molar ratio of BCC:*m*-XDA:BADGE was 1:1.15:0.3. Nanocomposite films with varying CNC contents were fabricated by adjusting the CNC loading. For CNC-W, loadings of 1, 2, 5, and 10 wt % (based on total solids) were used. In the case of CNC-T, loadings were limited to 1, 2, and 5 wt %, as higher contents resulted in poor dispersibility under the given conditions. The resulting PHU–epoxy/CNC nanocomposite films were designated as PHU–epoxy/CNC-W-1, PHU–epoxy/CNC-W-2, PHU–epoxy/CNC-W-5, PHU–epoxy/CNC-W-10, PHU–epoxy/CNC-T-1, PHU–epoxy/CNC-T-2, and PHU–epoxy/CNC-T-5.

### 2.5 Characterization

Atomic force microscopy (AFM) analysis was performed on a Multimode 8 AFM (Digital Instruments, USA) in ScanAsyst mode using a silicon cantilever probe with a tip radius of 2–12 nm and a resonance frequency of 70 kHz. A dilute aqueous CNC suspension (0.01 wt %) was drop-cast onto freshly cleaved mica substrates and allowed to dry under ambient conditions prior to imaging. X-ray diffraction (XRD) patterns were obtained using an X’Pert Pro diffractometer (model PW 3040/60) operating in the reflection mode at room temperature. The freeze-dried CNCs sample was pressed into pellets and scanned with rotation using a position-sensitive detector. Measurements were conducted with Cu Kα radiation (λ = 1.5418 Å), operated at 45 kV and 40 mA, and monochromatized with a 20 μm Ni filter. Diffraction patterns were recorded over a 2θ range of 5° to 50° at a scan rate of 2° min^–1^. The crystallinity index (CrI) of cellulose was calculated using the Segal method,^29^ according to the following equation:

1 $$CrI\%=\frac{\left(I_{200}-I_{amorphous}\right)}{I_{200}}\times 100$$

where *I*_200_ represents the maximum intensity of the (200) lattice diffraction peak at ∼22.7°, and *I*_*amorphous*_ is the intensity of the valley at ∼18°. The surface charge content of CNCs was determined via conductometric titration. Briefly, a suspension containing 150 mg of CNCs was dispersed in ultrapure water with the addition of 2 mL 0.1 M NaCl under continuous stirring to achieve a final total volume of 200 mL. The titration was carried out by incremental addition of standardized 0.01 M NaOH in 0.1 mL aliquots at 60 s intervals. Conductivity was monitored using a SevenCompact conductometric station (Mettler–Toledo). ^1^H NMR spectra were recorded to estimate monomer conversion during the synthesis of PHU prepolymers using a Bruker Avance III 400 MHz NMR spectrometer equipped with a direct cryoprobe at room temperature, with deuterated chloroform (CDCl_3_) used as the solvent. Fourier transform infrared (FTIR) spectroscopy was performed using a PerkinElmer Spectrum 2000 instrument equipped with an MKII Golden Gate single-reflection attenuated total reflectance (ATR) system (Specac Ltd., London, UK). The cross-sectional morphology of PHU–epoxy hybrid polymers and PHU–epoxy/CNC nanocomposite films was examined using a high-resolution, high-vacuum cold field-emission scanning electron microscope (FE-SEM, Hitachi S-4800) operated at accelerating voltages of 1 kV and 3 kV, a probe current of 10 μA, and a working distance of ∼8 mm. Samples were prepared by freeze-fracturing in liquid nitrogen and mounted on specimen holders. Prior to imaging, a ∼7 nm layer of gold–platinum was sputter-coated onto the fracture surfaces using an Au/Pt sputter coater (JFC1300). Differential scanning calorimetry (DSC) measurements were conducted using a TGA/DSC 1 STARe System (Mettler Toledo). Nitrogen was used as the purge gas at a flow rate of 50 mL min^–1^. Approximately 10 mg of sample was heated from −20 to 180 °C at a rate of 10 °C min^–1^, and data from the second heating cycle was used for analysis. Mechanical properties were evaluated using a universal testing machine (Instron 5944, UK) equipped with a 500 N load cell. Rectangular specimens (30 mm × 5 mm) were manually cut from the films. Prior to testing, the specimens were conditioned at 50% relative humidity (RH) and 22 °C for 48 h. Tensile tests were performed with a gauge length of 20 mm and a crosshead speed of 100 mm min^–1^. Five replicates were tested for each sample. Young’s modulus was determined from the slope of the initial linear region of the stress–strain curve, and toughness was defined as the work required to cause fracture, calculated as the area under the curve up to fracture. Dynamic mechanical analysis (DMA) was conducted using a TA Instruments Q800 in tension mode at a frequency of 1 Hz. The temperature was ramped from −30 to 180 °C for PHU–epoxy hybrid polymers and from −30 to 150 °C for PHU–epoxy/CNC nanocomposites, at a heating rate of 3 °C min^–1^.

## 3 Results and Discussion

### 3.1 Structure and Mechanical Properties of PHU–Epoxy Hybrid Polymer

The PHU–epoxy hybrid polymer was synthesized via a two-step process comprising the preparation of an amine-telechelic PHU prepolymer, followed by cross-linking through reaction with a diepoxide (Scheme 1). The monomer conversion of bis­(cyclic carbonates) was determined by ^1^H NMR (Figures S1–S5) and ranged from 93 to 95% for all formulations. Based on the measured cyclic carbonate conversion and the initial stoichiometry, the residual primary amine content in the prepolymers was estimated to increase from 0.17 to 0.56 mol equiv. relative to 1 mol of BCC as the BCC:m-XDA ratio increased from 1:1.10 to 1:1.50 (Table S1). The presence of residual amine groups confirms the formation of amine-terminated PHU prepolymers and provides reactive sites for subsequent epoxy curing. Figure 1 presents the FTIR spectra of the starting monomers BCC and *m*-XDA, the prepolymer, the curing agent BADGE, and the final PHU–epoxy hybrid polymer with a molar ratio of 1:1.15:0.3 (BCC:*m*-XDA:BADGE). Successful polymerization of BCC and *m*-XDA was evidenced by the ^1^H NMR and FTIR confirmed the disappearance of the characteristic carbonyl stretching band at 1781 cm^–1^, attributed to the cyclic carbonate group in BCC. Formation of the amine-terminated prepolymer was evidenced by the appearance of a weak band near 1589 cm^–1^, corresponding to the N–H bending vibration of primary amines (−NH_2_). Additionally, the emergence of a urethane carbonyl stretch at 1696 cm^–1^ and a broad band centered around 3300 cm^–1^, arising from overlapping O–H and N–H stretching vibrations, confirmed the formation of hydroxyurethane linkages. The broad absorption at 1696 cm^–1^, associated with hydrogen-bonded urethane carbonyl groups, was prominent, whereas signals in the 1710–1730 cm^–1^ range, characteristic of non-hydrogen-bonded carbonyls, were not observed.^30,31^ Moreover, the absence of a distinct band near 3500 cm^–1^ suggests that most N–H groups participate in hydrogen bonding.^32^

**Figure 1:**
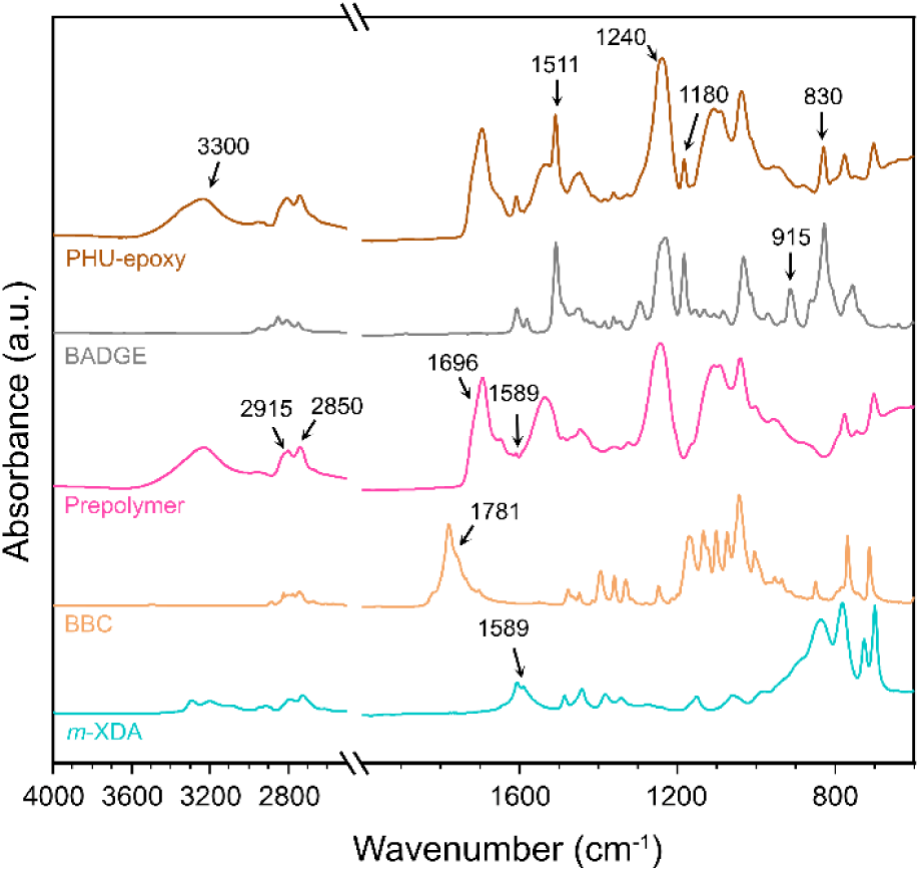
Stacked ATR-FTIR spectra of BCC, *m*-XDA, BADGE, the PHU prepolymer, and the PHU–epoxy hybrid polymer.

After cross-linking with BADGE, additional spectral changes were observed. In particular, further broadening of the band at ∼3300 cm^–1^ indicated an increase in hydroxyl content, consistent with epoxy ring opening by the amine groups of the prepolymer. The disappearance of the oxirane ring vibration at 915 cm^–1^ provided further evidence of epoxy–amine reaction. Additional peaks at 1511 cm^–1^, 1180–1240 cm^–1^, and 830 cm^–1^ were assigned to the aromatic C=C stretching, C–O, and C–O–C stretching vibrations, respectively, associated with BADGE incorporation and its para-substituted aromatic structure. Finally, the symmetric and asymmetric stretching vibrations of aliphatic C–H bonds in the polymer backbone were observed as a characteristic doublet at 2850 and 2915 cm^–1^.

The effect of chemical cross-linking on the mechanical properties of the PHU–epoxy hybrid polymer films with different epoxy contents was characterized by tensile test. Typical stress–strain curves are shown in Figure 2a, and the corresponding mechanical properties are summarized in Table 1. As the epoxy content increased from a BCC:*m*-XDA:BADGE ratio of 1:1.10:0.2 to 1:1.50:1.0, a pronounced enhancement in Young’s modulus was observed, rising from 1.5 to 604.2 MPa. A similar trend was found for tensile strength, which increased from 0.5 to 29.0 MPa. Conversely, the strain at break decreased significantly from 585.2% to 24.9%, reflecting increased stiffness and reduced ductility at higher epoxy content. These results indicate that epoxy content strongly influences the mechanical balance between strength and flexibility in the PHU–epoxy hybrid system. The formulation with a BCC:*m*-XDA:BADGE ratio of 1:1.15:0.30, exhibiting a toughness of 10.0 MJ m^–3^, was selected as the matrix for nanocomposite preparation with CNC-W and CNC-T. This composition provided a balanced set of properties, combining a moderate modulus (38.8 MPa) and tensile strength (4.5 MPa) with a high strain at break exceeding 360% and comparatively high toughness within the series. Selecting this intermediate formulation ensured sufficient structural integrity to reveal reinforcement effects of CNCs while avoiding the excessive brittleness of high-epoxy systems that could mask potential improvements.

**Figure 2:**
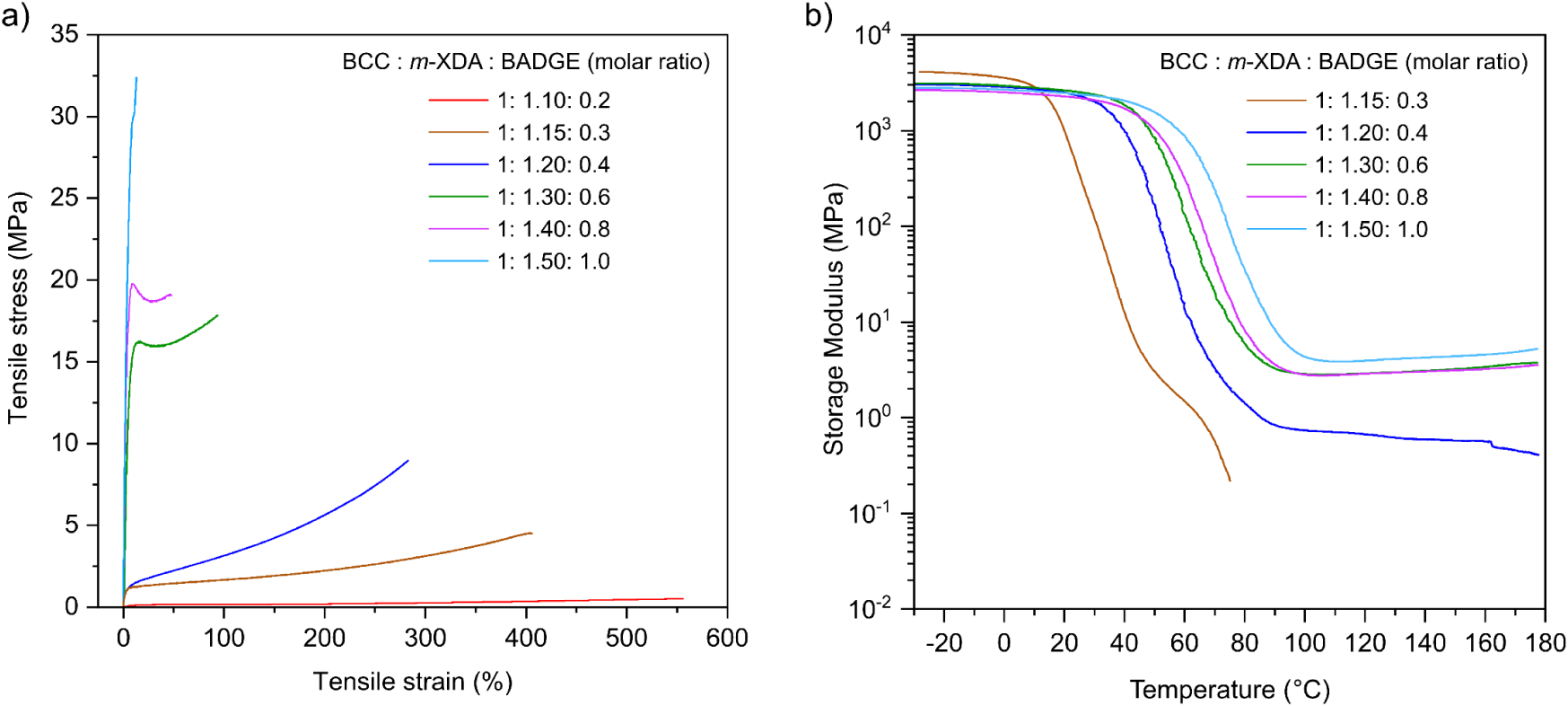
(a) Representative tensile stress–strain curves and (b) temperature-dependent storage modulus (*E′*) obtained by DMA of PHU–epoxy hybrid polymer films synthesized with different molar feed ratios of BCC, *m*-XDA, and BADGE (1:1.10:0.2 to 1:1.50:1.0).

**Table 1:** Mechanical Properties of PHU–Epoxy Hybrid Polymer Films Prepared with Different BCC:*M*-XDA:BADGE Molar Ratios, Determined by Tensile Testing^a^

Molar ratio	Young’s Modulus (MPa)	Tensile Strength (MPa)	Strain-at-break (%)	Toughness (MJ m^–3^)	Effective Cross-link Density (mol m^–3^)
1:1.10:0.2	1.5 ± 0.1	0.5 ± 0.2	585.2 ± 60.4	1.7 ± 0.6	-
1:1.15:0.3	38.8 ± 1.8	4.5 ± 0.5	362.9 ± 62.1	10.0 ± 1.7	-
1:1.20:0.4	37.4 ± 5.4	8.4 ± 0.6	275.3 ± 22.0	10.7 ± 1.5	60.4
1:1.30:0.6	387.5 ± 43.3	18.9 ± 1.7	99.2 ± 16.0	16.5 ± 4.1	314.8
1:1.40:0.8	487.4 ± 30.2	21.2 ± 1.4	66.0 ± 16.9	12.9 ± 5.0	309.4
1:1.50:1.0	604.2 ± 57.1	29.0 ± 3.1	24.9 ± 8.6	7.5 ± 0.8	433.4

a Effective cross-link density was estimated from DMA storage modulus values measured at 120 °C in the rubbery region using rubber elasticity theory.

In addition to tensile testing, DMA was performed to evaluate the thermomechanical behavior of the PHU–epoxy hybrid formulations (Figure 2b). The formulation with the lowest epoxy content could not be reliably analyzed, as the material was too soft and lacked sufficient dimensional stability to withstand oscillatory deformation over the selected temperature range. This observation indicates a very low effective cross-link density and limited network integrity. For formulations with higher epoxy content, the storage modulus (*E′*) values were comparable in the glassy region (approximately −30 to 20 °C), indicating similar stiffness at low temperatures. Pronounced differences emerged upon heating through the glass–rubber transition and into the rubbery regime. Increasing epoxy content led to a substantial increase in the rubbery plateau modulus, consistent with the formation of a more densely cross-linked and mechanically rigid network. In contrast, the formulation with a BCC:*m*-XDA:BADGE ratio of 1:1.15:0.30 showed the lowest modulus profile and a less pronounced rubbery plateau, reflecting reduced cross-link density and enhanced chain mobility. Although the highly cross-linked formulation displayed superior stiffness and strength, it maintained pronounced rigidity across the measured temperature range, confirming the restricted chain mobility inferred from tensile testing and accounting for its limited ductility. Excessive network stiffness would further obscure potential reinforcement effects arising from CNC incorporation, as the matrix response would already be dominated by high cross-link density.

The effective cross-link density (*ν*_*e*_) of the PHU-epoxy hybrid networks was estimated from the rubbery plateau storage modulus using classical rubber elasticity theory,^33–35^ according to the following equation:

2 $$\upsilon_{e}=\frac{E^{\prime }}{3RT}$$

where *E′* is the storage modulus in the rubbery region, *R* is the universal gas constant (8.314 J mol^–1^·K^–1^), and *T* is the absolute temperature (K). Effective cross-link density was estimated using storage modulus values measured at 120 °C (393 K). This temperature was selected because it lies within the rubbery region of the measurable formulations and sufficiently above the glass transition temperature (*T*_*g*_) to enable comparison among samples. The calculated values are summarized in Table 1. It should be noted that *ν*_*e*_ represents an effective network density derived from rubber elasticity theory and therefore reflects both covalent cross-links and restrictions on chain mobility within the network.

The effective cross-link density increased substantially with increasing epoxy content, from 60.4 mol m^–3^ for the 1:1.20:0.4 formulation to 433.4 mol m^–3^ for the 1:1.50:1.0 formulation. This trend is consistent with the progressive increase in rubbery plateau modulus and supports the formation of increasingly constrained network structures at higher BADGE contents. In contrast, the formulation with a BCC:m-XDA:BADGE ratio of 1:1.15:0.30 did not exhibit a well-defined rubbery plateau over the investigated temperature range, preventing reliable estimation of *ν*_*e*_ and suggesting a lower effective network density and greater chain mobility. These results are in good agreement with the tensile properties and further support the selection of the intermediate formulation as a balanced matrix for subsequent CNC reinforcement studies.

### 3.2 Structure of PHU–Epoxy/CNC Nanocomposites

CNC-T was successfully prepared via sulfuric acid hydrolysis of tunicate-derived cellulose. Its morphology was characterized by AFM and compared with that of the commercial CNC-W derived from wood cellulose, as shown in Figure S6 (Supporting Information). AFM height images revealed that both CNC-T and CNC-W exhibit the characteristic needle-like morphology. However, CNC-W showed significantly shorter nanocrystals, while CNC-T consisted of markedly longer nanocrystals. To quantitatively assess their differences, dimensional measurements were performed on over 300 individual nanocrystals using ImageJ software. CNC-W showed lengths ranging from 70 to 330 nm and widths from 4 to 14 nm. In contrast, CNC-T showed lengths ranging from 390 to 1825 nm and widths from 3 to 26 nm. Based on the peak values of the measured length and width distributions, the aspect ratio (length-to-width) of CNC-T was calculated to be ∼61, significantly higher than that of CNC-W (∼19). The crystallinity and crystal structures of CNC-T and CNC-W were analyzed by XRD (Figure S7, Supporting Information). Both CNC types exhibited characteristic diffraction peaks at 2θ values of 14.7°, 16.4°, 22.7°, and 34.5°, corresponding to the (11̅0), (110), (200), and (004) crystallographic planes of cellulose I, respectively.^36–38^ The CrI was 82% for CNC-W and 90% for CNC-T, indicating a slightly higher degree of structural order in CNC-T. The sulfate half-ester content, determined by conductometric titration, was 0.17 mmol g^–1^ for CNC-W and 0.15 mmol g^–1^ for CNC-T.

To prepare the nanocomposites, different amounts of CNCs were incorporated into the BCC/*m*-XDA prepolymer together with BADGE. The FTIR spectra (Figure 3) revealed the disappearance of the band at 1781 cm^–1^ corresponding to the C=O stretching vibrations of the cyclic carbonate and the appearance of a urethane carbonyl stretching band at 1696 cm^–1^ in all nanocomposites, indicating successful ring-opening polymerization and formation of PHU linkages. In particular, no distinct peaks were observed in the 1710–1730 cm^–1^ region, characteristic of non-hydrogen-bonded carbonyl groups,^30,31^ suggesting that most carbonyl groups participate in hydrogen bonding in the PHU–epoxy/CNC nanocomposites. The broad absorption band in the range of 3200–3500 cm^–1^, assigned to overlapping N–H and O–H stretching vibrations, became increasingly pronounced and broadened with increasing CNCs content in both PHU–epoxy/CNC-W and PHU–epoxy/CNC-T nanocomposites. The absence of a distinct band near 3500 cm^–1^ suggests that N–H groups remain largely engaged in hydrogen-bonding.^32^ These observations are consistent with strong intermolecular hydrogen bonding between PHU and CNC hydroxyl groups. The increase in the 1240 cm^–1^ and 1033 cm^–1^ bands with CNC loading is also consistent with the growing contribution of cellulose C–O and C–O–C stretching vibrations in the nanocomposites.

**Figure 3:**
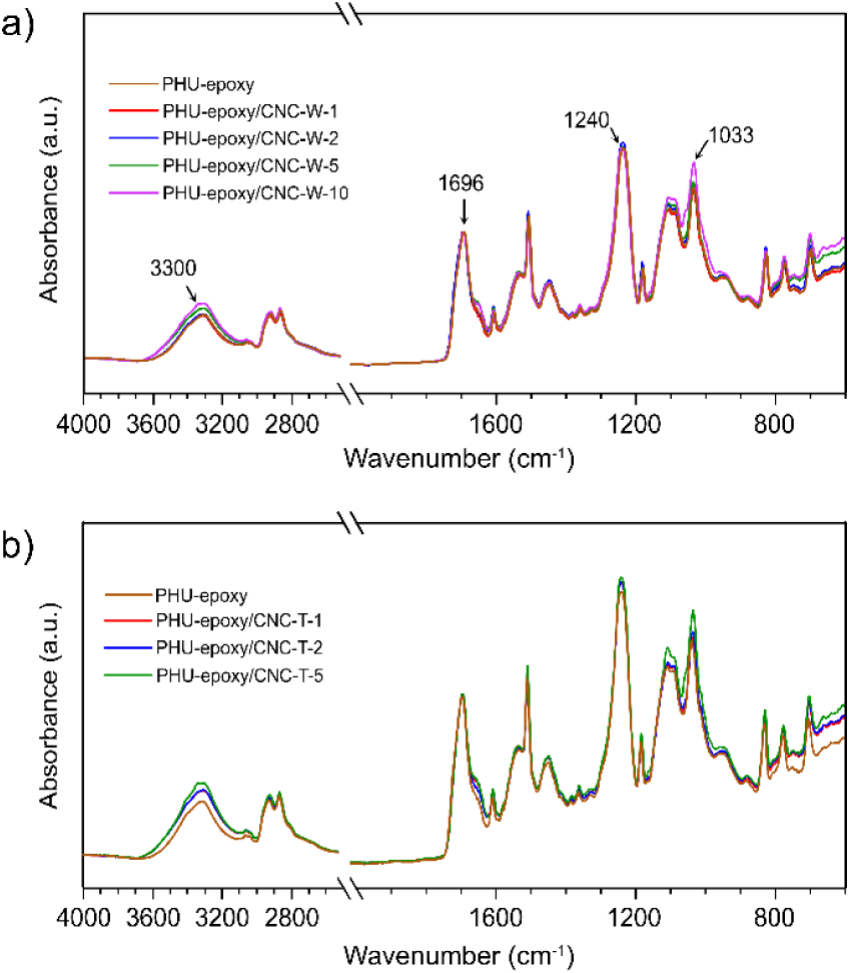
Overlaid ATR-FTIR spectra of (a) PHU–epoxy/CNC-W and (b) PHU–epoxy/CNC-T nanocomposites with varying CNC loadings, compared to the neat PHU–epoxy matrix.

The prepared PHU–epoxy hybrid polymer film exhibited high optical transparency, whereas incorporation of CNCs led to a gradual decrease in transparency, with the nanocomposites becoming slightly translucent at higher CNC loadings (Figure S8, Supporting Information). To evaluate CNC dispersion within the PHU–epoxy matrix, the nanocomposite morphology was characterized by FE-SEM. Representative cryo-fractured cross-sectional surfaces of neat PHU–epoxy and the nanocomposite films PHU–epoxy/CNC-T-5 and PHU–epoxy/CNC-W-10 are shown in Figure 4. The neat PHU–epoxy film exhibited a relatively smooth and uniform surface, while the nanocomposites displayed rougher fracture surfaces due to the incorporation of crystalline CNCs. Overall, the CNCs appear well dispersed and homogeneously distributed throughout the matrix. However, minor aggregates consisting of a few CNC crystallites were also observed in both types of nanocomposites.

**Figure 4:**
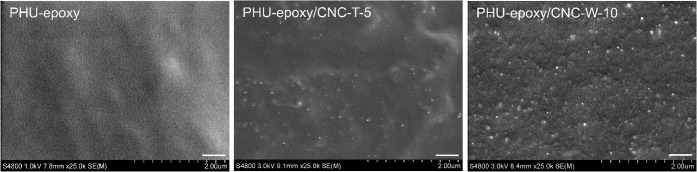
FE-SEM images of freeze-fractured cross-sectional surfaces of PHU–epoxy hybrid polymer, PHU–epoxy/CNC-T-5, and PHU–epoxy/CNC-W-10 nanocomposite films (scale bar: 500 nm).

### 3.3 Thermal and Mechanical Properties of PHU–Epoxy/CNC Nanocomposites

The thermal properties of the nanocomposites were investigated by DSC. The second-heating DSC thermograms (Figure 5) revealed a single, broad glass transition at 20 °C for neat PHU-epoxy and all nanocomposite samples. For all PHU–epoxy/CNC-T nanocomposites and for the PHU–epoxy/CNC-W nanocomposites at lower filler contents (≤2 wt %), no significant change in the glass transition temperature (*T*_*g*_) was observed (Table 2). This behavior is consistent with previous reports, in which *T*_*g*_ of nanocellulose-reinforced systems typically shows little variation or a slight decrease upon filler incorporation,^39,40^ particularly in epoxy-^41^ and gluten-based composites.^42–44^ In contrast, at higher CNC-W loadings (≥5 wt %), a slight increase in *T*_*g*_ was detected. This increase is commonly attributed to reduced chain mobility near the filler–matrix interface, arising from interfacial interactions between CNCs and the polymer matrix. The incorporation of higher amounts of CNC-W, which possess a lower aspect ratio and consequently a larger number of dispersed particles at equivalent mass fraction, may increase the interfacial area available for hydrogen bonding between CNC surfaces and the PHU–epoxy network, thereby restricting segmental dynamics.^45^

**Figure 5:**
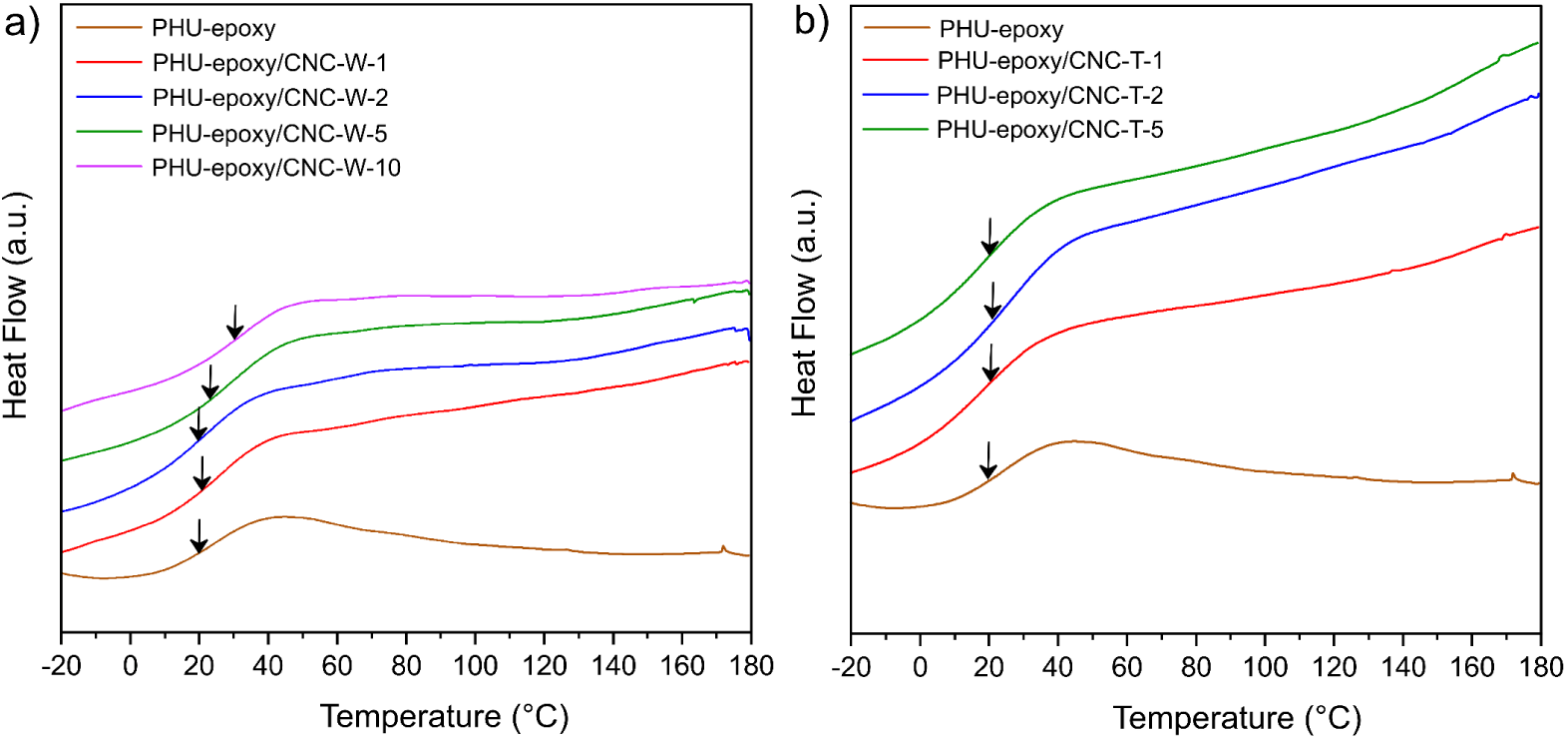
Second-heating DSC thermograms of (a) PHU–epoxy/CNC-W and (b) PHU–epoxy/CNC-T nanocomposites with varying CNC loadings, compared with the neat PHU–epoxy matrix. Arrows indicate the glass transition temperature (*T_g_*). The corresponding *T_g_* values are summarized in Table 2.

**Table 2:** Summary of the Mechanical and Thermomechanical Properties of PHU–Epoxy /CNC-W and PHU–Epoxy/CNC-T Nanocomposites with Varying CNC Contents^a^

Sample	Young’s modulus (MPa)	Tensile strength (MPa)	Strain-at-break(%)	Toughness (MJ m^–3^)	Effective Cross-link Density (mol m^–3^)	*T*_g_ (°C, DSC)
PHU–epoxy/CNC-W-1	40.0 ± 5.7	6.3 ± 1.0	366.0 ± 36.1	11.7 ± 1.1	-	20
PHU–epoxy/CNC-W-2	34.0 ± 7.8	10.1 ± 1.5	377.2 ± 20.8	16.4 ± 1.2	62.6	19
PHU–epoxy/CNC-W-5	52.9 ± 7.4	10.7 ± 0.4	282.9 ± 14.9	15.6 ± 0.1	126.0	21
PHU–epoxy/CNC-W-10	125.1 ± 33.4	11.7 ± 0.6	261.2 ± 18.3	17.0 ± 0.2	425.5	24
PHU–epoxy/CNC-T-1	182.8 ± 23.5	11.8 ± 0.3	313.5 ± 42.7	26.6 ± 0.1	101.8	20
PHU–epoxy/CNC-T-2	274.3 ± 22.1	12.5 ± 0.2	244.0 ± 12.4	27.2 ± 1.3	193.5	20
PHU–epoxy/CNC-T-5	294.8 ± 35.6	15.1 ± 0.7	121.4 ± 17.6	16.1 ± 4.4	1364.4	19

a Mechanical properties were obtained from tensile testing. Effective cross-link density was estimated from DMA storage modulus values measured at 120 °C in the rubbery region using rubber elasticity theory, and *T_g_* was determined by DSC.

Thermogravimetric analysis (Figure S9, Supporting Information) showed that CNC incorporation had only a minor effect on the thermal stability of the PHU–epoxy matrix. All nanocomposites exhibited degradation profiles similar to that of neat PHU–epoxy. A slight increase in thermal stability was observed for the CNC-T nanocomposites, which may be attributed to the higher crystallinity of tunicate-derived CNCs. Overall, the results indicate that CNC reinforcement primarily affects the mechanical and thermomechanical properties of the hybrid network while having limited influence on its thermal degradation behavior.

Figure 6 shows representative stress–strain curves of the PHU–epoxy/CNC nanocomposites, and the corresponding mechanical properties are summarized in Table 2. The neat PHU–epoxy hybrid polymer exhibited distinct deformation stages, including a subtle yield point, plastic deformation, and strain hardening, ultimately reaching a tensile strength of 4.5 MPa and a strain at break of 362.9%, behavior characteristic of thermoplastic polyurethanes (TPUs).^46^ All nanocomposites, except PHU–epoxy/CNC-T-5, showed similar transitions, with an initial linear region up to 5–10% strain followed by yielding and pronounced strain hardening at higher elongations until fracture. Notably, most nanocomposites exhibited a stronger upward curvature in the stress–strain curves, indicating enhanced strain hardening and higher ultimate tensile strength without a severe loss of strain-at-break. In contrast, PHU–epoxy/CNC-T-5 showed a more brittle response, fracturing without significant strain hardening.

**Figure 6:**
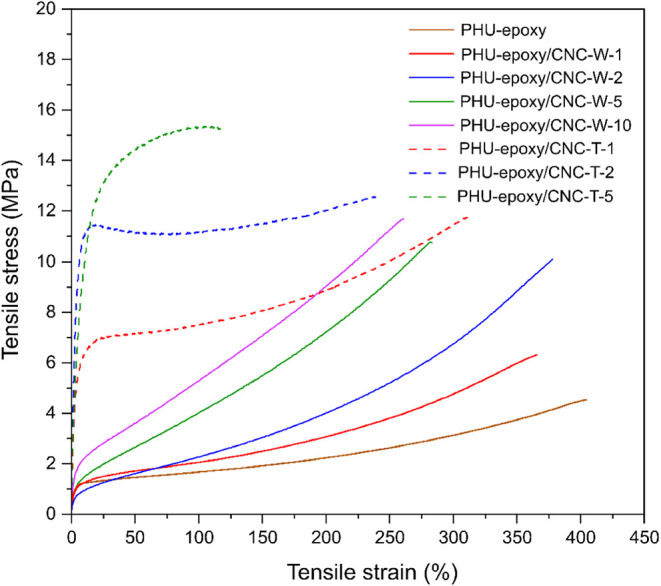
Representative tensile stress–strain curves of the PHU–epoxy/CNC-W and PHU–epoxy/CNC-T nanocomposites at various CNC loadings, compared with the neat PHU–epoxy matrix.

The incorporation of CNCs substantially enhanced both Young’s modulus and tensile strength. The modulus increased from 38.8 MPa for neat PHU–epoxy to 125.1 MPa for PHU–epoxy/CNC-W-10, reflecting the reinforcing effect of dispersed CNC-W. This improvement is attributed to hydrogen bonding between hydroxyl groups on CNC-W surfaces and urethane groups within the PHU–epoxy matrix. Owing to their lower aspect ratio and consequently higher number density at a given mass fraction, CNC-W provides numerous filler–matrix interaction sites, consistent with the slight *T*_*g*_ increase observed at higher CNC-W contents. For CNC-T, even at low loadings, reinforcement was more pronounced. The modulus of PHU–epoxy/CNC-T-5 reached 294.8 MPa while the tensile strength increased to 15.1 MPa. This superior reinforcement is associated with the higher aspect ratio of CNC-T, which promotes the formation of an interconnected CNC network structure capable of more efficient stress transfer within the matrix.^27,47,48^ Such structural organization enhances load-bearing capacity and contributes to the significant improvement in mechanical performance.^49^ The modulus values reported here exceed those of conventional PU/CNC nanocomposites (e.g., 44.9 MPa at 5 wt % CNC and 101 MPa at 20 wt % CNC reported by Pei et al.^32^ and Redondo et al.,^50^ respectively), as well as PHU composites reinforced with surface modified CNCs (75 MPa at 10 wt % poly­(*N*-vinylpyrrolidone)-modified CNC reported by Ge et al.^51^) or superhydrophobic silica nanoparticles (2.7 MPa at 4 wt % silica reported by Chen et al.^17^).

As expected, the strain at break decreased with increasing CNCs loading. Nevertheless, PHU–epoxy/CNC-W nanocomposites retained strain-at-break values above 250% even at 10 wt % CNC loading, demonstrating the ability to maintain substantial ductility despite increased stiffness. In contrast, PHU–epoxy/CNC-T nanocomposites exhibited a more pronounced reduction in strain-at-break value. For both systems, stiffness increased with CNC content, as reflected by the steeper slope in the elastic region. Importantly, even at higher CNC contents, the nanocomposites avoided excessive stiffening and preserved considerable strain-at-break compared with highly cross-linked PHU–epoxy hybrids. The toughness of PHU–epoxy/CNC-T nanocomposites was higher than that of PHU–epoxy/CNC-W nanocomposites at moderate loadings. However, toughness declined at the highest CNC-T loading (5 wt %) due to reduced ductility. As toughness reflects the balance between strength and elongation, increasing CNC content ultimately led to stronger but more brittle materials.

The thermomechanical properties of the PHU–epoxy/CNC nanocomposites were evaluated by DMA, and the results are shown in Figure 7. At −30 °C, all samples were in the glassy state. For neat PHU–epoxy, a pronounced decrease in storage modulus was observed during the glass–rubber transition, followed by a gradual decline in the rubbery region (Figure 7a). In contrast, the nanocomposites exhibited a reduced drop in storage modulus upon CNC incorporation and reached a more stable plateau at elevated temperatures. The reinforcing effect of CNCs was particularly evident above 80 °C, the storage modulus of PHU–epoxy/CNC-W-10 and PHU–epoxy/CNC-T-5 exceeded that of neat PHU–epoxy by approximately 200- and 300-fold, respectively. This enhancement is attributed to the formation of an interconnected CNC structure within the matrix, stabilized by hydrogen bonding interactions. The higher modulus observed for PHU–epoxy/CNC-T compared to PHU–epoxy/CNC-W nanocomposites is likely related to the higher aspect ratio and elongated morphology of CNC-T, which facilitate network formation and more efficient stress transfer at lower loadings. These results suggest that reinforcement in PHU–epoxy/CNC-T nanocomposites is increasingly governed by filler–filler interactions as CNC content increases, whereas in PHU–epoxy/CNC-W systems, filler–matrix interactions appear to play a more dominant role. Although conventional composite design often emphasizes strong filler–matrix adhesion for optimal performance,^52^ CNC-based nanocomposites can exhibit significant reinforcement through the development of interconnected filler structures.^52,53^

**Figure 7:**
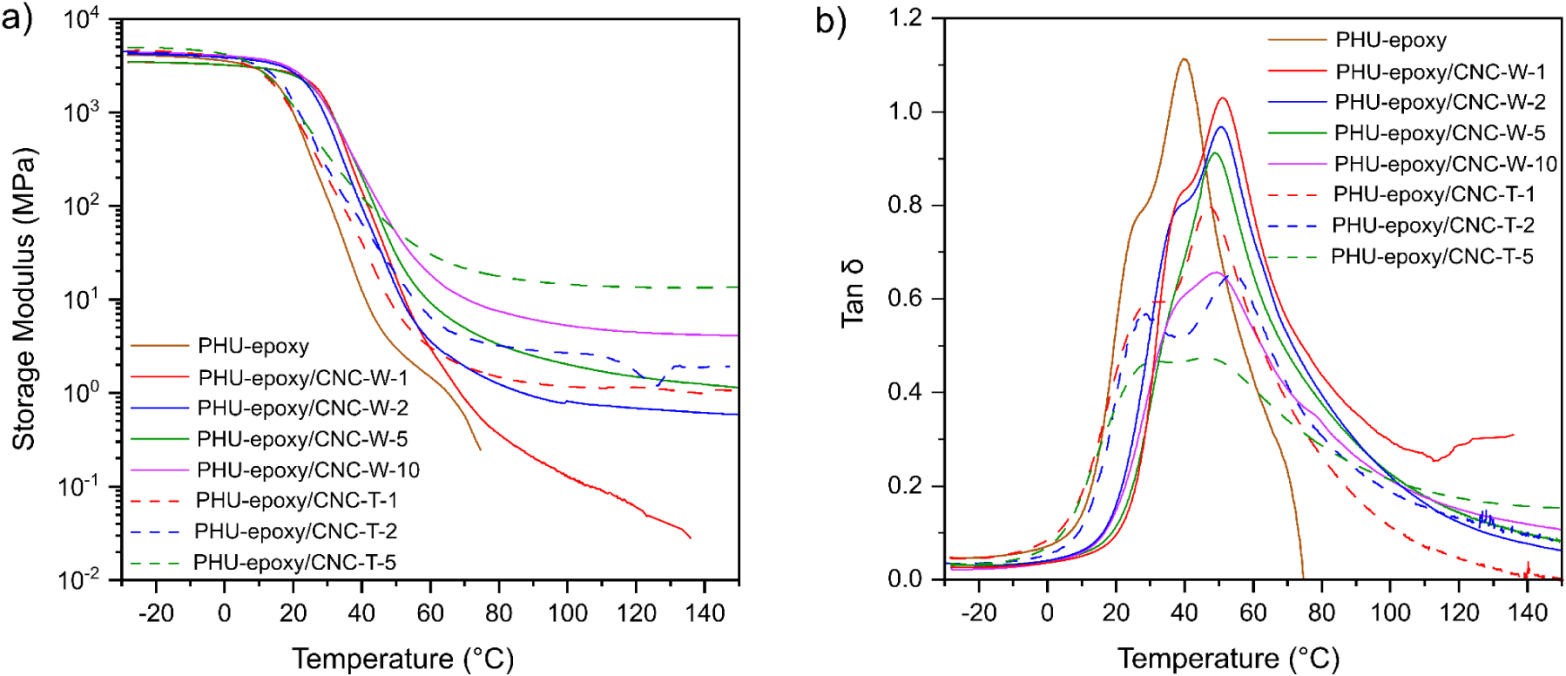
(a) Storage modulus (*E′*) and (b) tan δ as a function of temperature for PHU–epoxy/CNC-W and PHU–epoxy/CNC-T nanocomposites at various CNC loadings, compared with the neat PHU–epoxy matrix.

The effective cross-link density (*ν*_*e*_) of the PHU–epoxy/CNC nanocomposites was estimated from the rubbery plateau storage modulus using classical rubber elasticity theory, and the calculated values are summarized in Table 2. Similar to the neat hybrid system, the calculated *ν*_*e*_ represents an effective network density that incorporates contributions from both covalent cross-links and physical interactions. With increasing CNC content, an apparent increase in *ν*_*e*_ was observed for both CNC-W and CNC-T systems. This increase does not arise from additional chemical cross-linking, but rather from restricted chain mobility and the formation of physical junctions associated with hydrogen bonding and polymer–CNC interfacial interactions.

The effect was more pronounced for the PHU–epoxy/CNC-T nanocomposites, which is consistent with the higher aspect ratio of CNC-T and its greater tendency to form interconnected filler structures within the matrix. Such structures contribute to the elastic response in the rubbery region and therefore increase the apparent cross-link density estimated from DMA. The exceptionally high *ν*_*e*_ value (1364.4 mol m^–3^) estimated for PHU–epoxy/CNC-T-5 should therefore be interpreted as reflecting the strong contribution of the interconnected CNC structure to the rubbery modulus rather than a corresponding increase in covalent cross-link density. In contrast, the more moderate increase observed for the CNC-W nanocomposites suggests that reinforcement is governed primarily by filler–matrix interactions rather than extensive filler–filler connectivity. These results further support the interpretation that the thermomechanical behavior of the nanocomposites is controlled by a combination of the polymer network structure and nanofiller-induced physical constraints.

The tan δ curves (Figure 7b) revealed a shoulder at lower temperature for all samples, indicative of multiple mechanical relaxation processes not resolved in DSC. This feature may arise from variations in segmental mobility, cross-link density, and network heterogeneity.^45,54^ For the PHU–epoxy/CNC-W nanocomposites, the tan δ peak shifted to higher temperatures, reflecting restricted chain mobility caused by hydrogen bonding between CNC-W and PHU–epoxy matrix.^55^ This observation is consistent with the *T*_*g*_ increase detected by DSC at higher CNC-W contents. The incorporation of CNC-W and CNC-T resulted in a maximum *T*_*g*_ increase of approximately 14 °C, as determined from the tan δ maximum. The damping behavior provides additional insight into molecular mobility within the matrix.^45,56,57^ A reduction in tan δ intensity indicates a more elastic response with lower energy dissipation during mechanical deformation.^58^ In PHU–epoxy/CNC-T nanocomposites, the T_*g*_-associated tan δ peak decreased in intensity and broadened with increasing CNC-T content, suggesting increasing structural heterogeneity and restricted chain mobility. This effect was less pronounced for PHU–epoxy/CNC-W nanocomposites, consistent with the different reinforcement mechanisms inferred from mechanical testing.

### 3.4 Comparison of Epoxy-Driven Hybridization and CNC-Driven Reinforcement

Previous studies have demonstrated that incorporation of nanocellulose into PHU systems can improve mechanical performance; however, these approaches often rely on surface modification of the nanofillers or focus on systems where matrix structure is not systematically controlled.^51^ For instance, recent work on PHU/CNC nanocomposites has shown improved dispersion and reinforcement at higher loadings, while highlighting the importance of interfacial interactions in governing final properties.^23^ In epoxy-based nanocomposites, CNC incorporation can enhance stiffness; however, effective reinforcement depends strongly on dispersion and filler–matrix interactions, often requiring surface modification to improve compatibility and stress transfer.^59^

In our previous work, segmented PHU systems reinforced with CNC-W and chitin nanocrystals were investigated to elucidate how interfacial interactions and bonding characteristics influence composite performance.^22^ Building on these findings, the present study focuses on PHU–epoxy hybrid networks in which the matrix structure is systematically tuned through covalent cross-linking prior to CNC incorporation. This approach enables a direct comparison between matrix hybridization and nanofiller reinforcement while identifying compositions that balance stiffness, strength, ductility, and toughness. Furthermore, by comparing CNCs with different aspect ratios, this work provides additional insight into how nanocrystal morphology influences reinforcement efficiency in hybrid PHU systems.

In PHU–epoxy hybrids, increasing epoxy content (Table 1 and Figure 2) produces a pronounced rise in Young’s modulus (from 1.5 to 604 MPa) and tensile strength (from 0.5 to 29 MPa), accompanied by a substantial reduction in strain at break (from 585% to 25%), consistent with increasing covalent cross-linking. Toughness follows a nonmonotonic trend, reaching a maximum of ∼16.5 MJ m^–3^ at intermediate epoxy content (1:1.30:0.6) before declining at the highest epoxy level as brittleness increases. This behavior indicates that stiffness enhancement through cross-linking eventually outweighs the material’s ability to dissipate energy through plastic deformation. The DMA results of the PHU–epoxy hybrids (Figure 2b) further support this trend, showing a systematic increase in rubbery plateau modulus with increasing epoxy content.

When CNC reinforcement is introduced into the intermediate PHU–epoxy matrix with a BCC:*m*-XDA:BADGE ratio of 1:1.15:0.3 (Table 2 and Figure 6), substantial improvements in modulus and strength are obtained at relatively low filler loadings while preserving considerable ductility. This effect is particularly evident for CNC-W, which maintains a strain-at-break above 260% even at 10 wt % loading. CNC-T provides stronger reinforcement at lower contents. At 5 wt % CNC-T loading, the modulus increases to ∼295 MPa and the tensile strength reaches ∼15 MPa. Importantly, the highest toughness is achieved at moderate CNC-T loadings of 1–2 wt %, reaching ∼26.6–27.2 MJ m^–3^. These values exceed those of CNC-W nanocomposites, which reach up to 17 MJ m^–3^ at 10 wt %, as well as PHU–epoxy hybrids, which peak at 16.5 MJ m^–3^. At 5 wt % CNC-T, toughness decreases to ∼16 MJ m^–3^, accompanied by embrittlement and the disappearance of strain hardening. This behavior indicates a shift toward stiffness-dominated mechanical response at higher filler contents.

To place these results in a broader context, the reinforcement efficiency of the present system was compared with selected PHU- and PU-based nanocomposites reported in the literature (Figure 8). While previous PHU/CNC systems often required surface-modified nanocellulose or higher filler loadings to achieve moderate stiffness enhancement, and conventional PU/CNC systems frequently showed reinforcement strongly dependent on dispersion and interfacial compatibility, the present PHU–epoxy/CNC system demonstrates a pronounced increase in modulus at relatively low filler contents. In particular, CNC-T provides substantial reinforcement at low loadings, highlighting the influence of high-aspect-ratio nanocrystals on reinforcing efficiency. The combination of a covalently cross-linked PHU–epoxy matrix and well-dispersed CNCs enables stiffness levels that exceed those reported for several PHU- and PU-based nanocomposites at comparable or higher filler contents.

**Figure 8:**
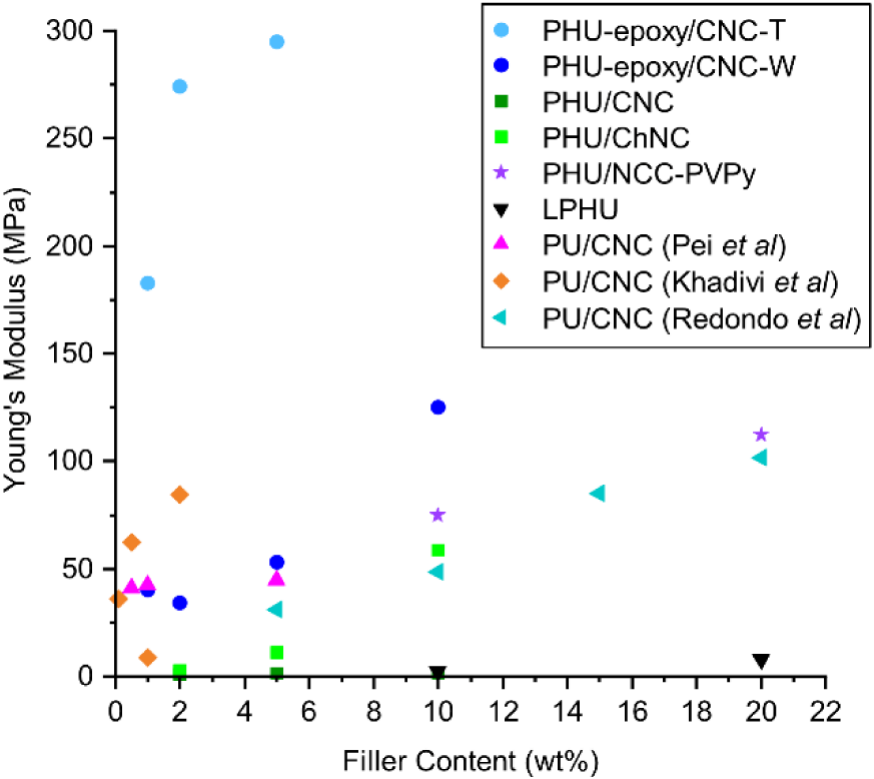
Young’s modulus versus filler content for PHU-epoxy hybrids reinforced with CNC-T and CNC-W (this work), segmented PHU reinforced with CNC-W and chitin nanocrystals (ChNC),^22^ PHU reinforced with surface modified CNC (NCC-PVPy),^51^ PHU reinforced with lignin (LPHU),^17^ and PU reinforced with CNC.^32,50,60^

FTIR analysis indicates extensive hydrogen bonding among PHU segments, epoxy-derived linkages, and CNC surfaces, while SEM reveals generally homogeneous dispersion with only minor aggregation. For CNC-W, reinforcement appears to be governed primarily by filler–matrix interactions, as reflected in the modest *T*_*g*_ increase and preservation of ductility. In contrast, the higher aspect ratio of CNC-T facilitates the formation of an interconnected filler structure at lower loadings, leading to pronounced enhancement of high-temperature storage modulus (DMA: ∼300-fold increase at 5 wt % CNC-T compared with ∼200-fold at 10 wt % CNC-W) and superior stiffness at relatively low filler content. The maximum toughness observed at 1–2 wt % CNC-T likely arises from a favorable balance between efficient stress transfer within the interconnected CNC-T framework and sufficient matrix deformability to enable energy dissipation through reversible hydrogen-bond rearrangement and polymer chain mobility. At higher CNC-T loadings, reduced matrix continuity and restricted chain mobility limit energy absorption, resulting in embrittlement.

Overall, epoxy-driven hybridization effectively establishes baseline stiffness and strength but provides limited gains in toughness beyond intermediate cross-link densities. CNC reinforcement, particularly using high-aspect ratio tunicate-derived CNCs, enables greater stiffness per unit loading and achieves superior toughness at moderate filler contents. Thus, combining controlled epoxy content with appropriate CNC type and loading offers a strategy to simultaneously enhance modulus and toughness while maintaining usable ductility in PHU-based materials. The simultaneous improvement in stiffness and toughness observed for the PHU–epoxy/CNC nanocomposites is particularly relevant for applications such as coatings, adhesives, and sustainable composite matrices, where resistance to deformation must be balanced with resistance to crack initiation and propagation. In such applications, materials that combine load-bearing capability with high energy absorption are often preferred over systems optimized solely for stiffness or strength. In addition, recent studies have shown that PHU–epoxy hybrid networks can exhibit dynamic covalent behavior enabling stress relaxation and reprocessability.^12^ In this context, it would be of interest in future work to examine how the combined effects of covalent cross-linking and CNC-induced hydrogen-bonding interactions influence these dynamic properties in the present system.

## 4 Conclusion

PHU–epoxy hybrids and PHU–epoxy/CNC nanocomposites represent complementary strategies for enhancing the mechanical performance of PHU materials. Increasing epoxy content establishes a higher baseline modulus and strength but progressively reduces extensibility, with toughness reaching a maximum at intermediate epoxy content (∼16.5 MJ m^–3^) and declining at high cross-link densities. Reinforcement of an intermediate PHU–epoxy matrix with CNCs provides substantial stiffness enhancement at low filler loadings. In particular, tunicate-derived CNCs achieve the highest toughness in this study (∼27 MJ m^–3^ at 2 wt %), surpassing both wood-derived CNC nanocomposites and all PHU–epoxy hybrids. DMA further reveals improved high-temperature stiffness retention for tunicate-derived CNC systems, which is associated with the formation of an interconnected high–aspect ratio filler structure. In contrast, wood-derived CNCs exhibit stronger matrix interactions and better preserve ductility at higher filler loadings. These results demonstrate that controlled epoxy hybridization combined with appropriate CNC selection and loading enables simultaneous enhancement of modulus, strength, and toughness in biobased PHU materials.

## Supplementary Material



## Notes

^1^H NMR spectra of the BCC:m-XDA PHU prepolymer samples; AFM height images, width and length distributions, and XRD pattern of CNCs; and photographs and TGA curves of the PHU–epoxy/CNC nanocomposites (PDF)
