# Supplementary material for: Toughening of Poly(hydroxyurethane)–Epoxy Hybrid Networks by High-Aspect-Ratio Cellulose Nanocrystals
Source: Biomacromolecules. 2026 Jul 14;27(8):5318–29. doi: 10.1021/acs.biomac.6c00531 (PMC13463539; doi:10.1021/acs.biomac.6c00531)
Supplement: Supplementary file 1 [file bm6c00531_si_001.pdf]

Supporting Information

Toughening of Poly(hydroxyurethane)–Epoxy Hybrid Networks by High-Aspect-Ratio Cellulose Nanocrystals

Pavithra M. Wijeratne, [†,‡] *Li Zha,* [†] *Connie Ocando,* [‡] *Lars A. Berglund,* [§] *Jean-Marie Raquez,* [‡] *and Qi Zhou*[*,†]

[†] Division of Glycoscience, Department of Chemistry, School of Engineering Sciences in Chemistry, Biotechnology and Health, KTH Royal Institute of Technology, AlbaNova University Centre, 106 91 Stockholm, Sweden

[‡] Laboratory of Polymeric and Composite Materials, Department of Chemistry, Faculty of Science, University of Mons, 7000 Mons, Belgium

[§] Department of Fiber and Polymer Technology, KTH Royal Institute of Technology, 100 44 Stockholm, Sweden

E-mail: qi@kth.se

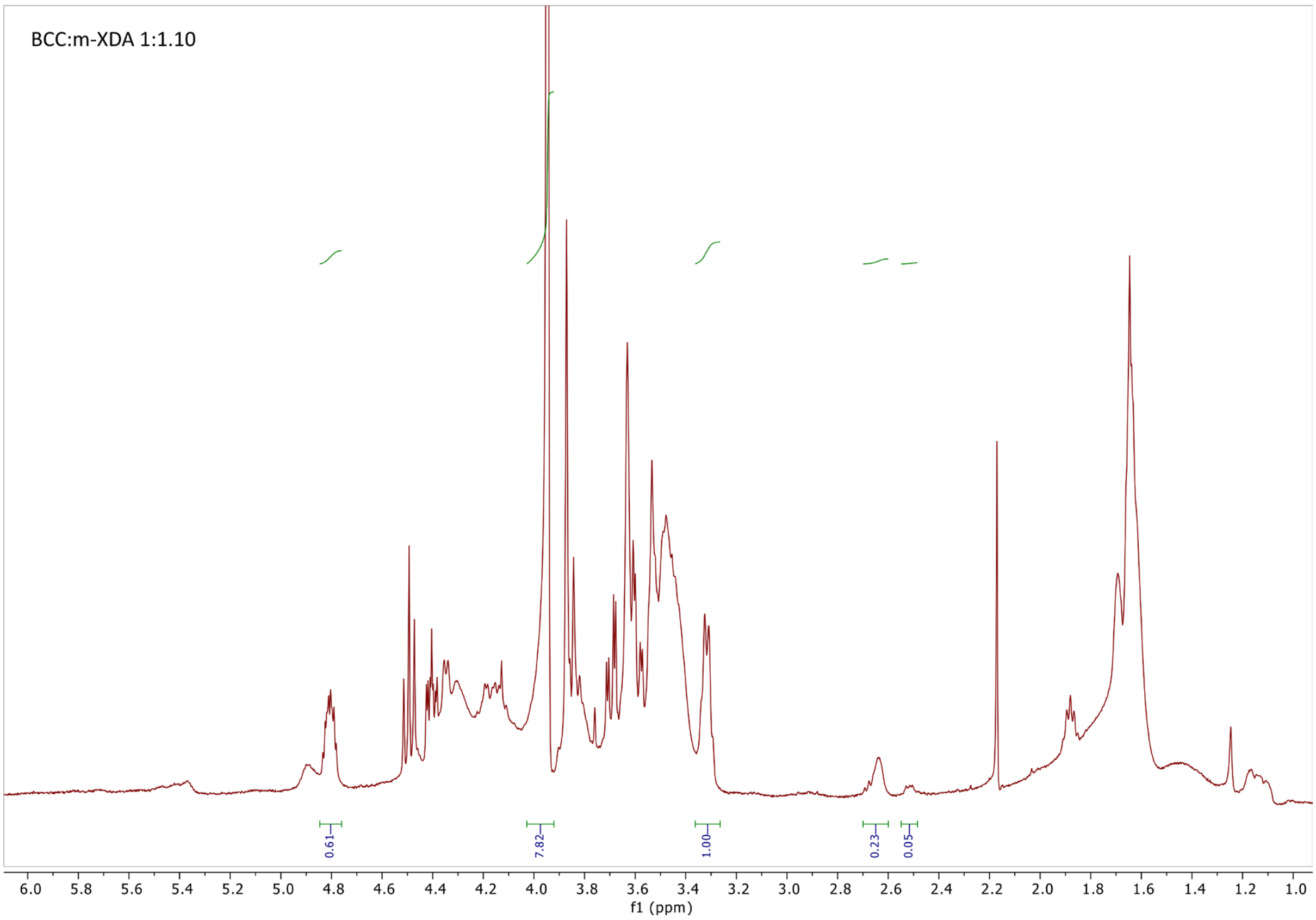

Figure S1. ^{1}H NMR Spectrum of the BCC:m-XDA 1:1.10 PHU pre-polymer in $CDCl_3$.

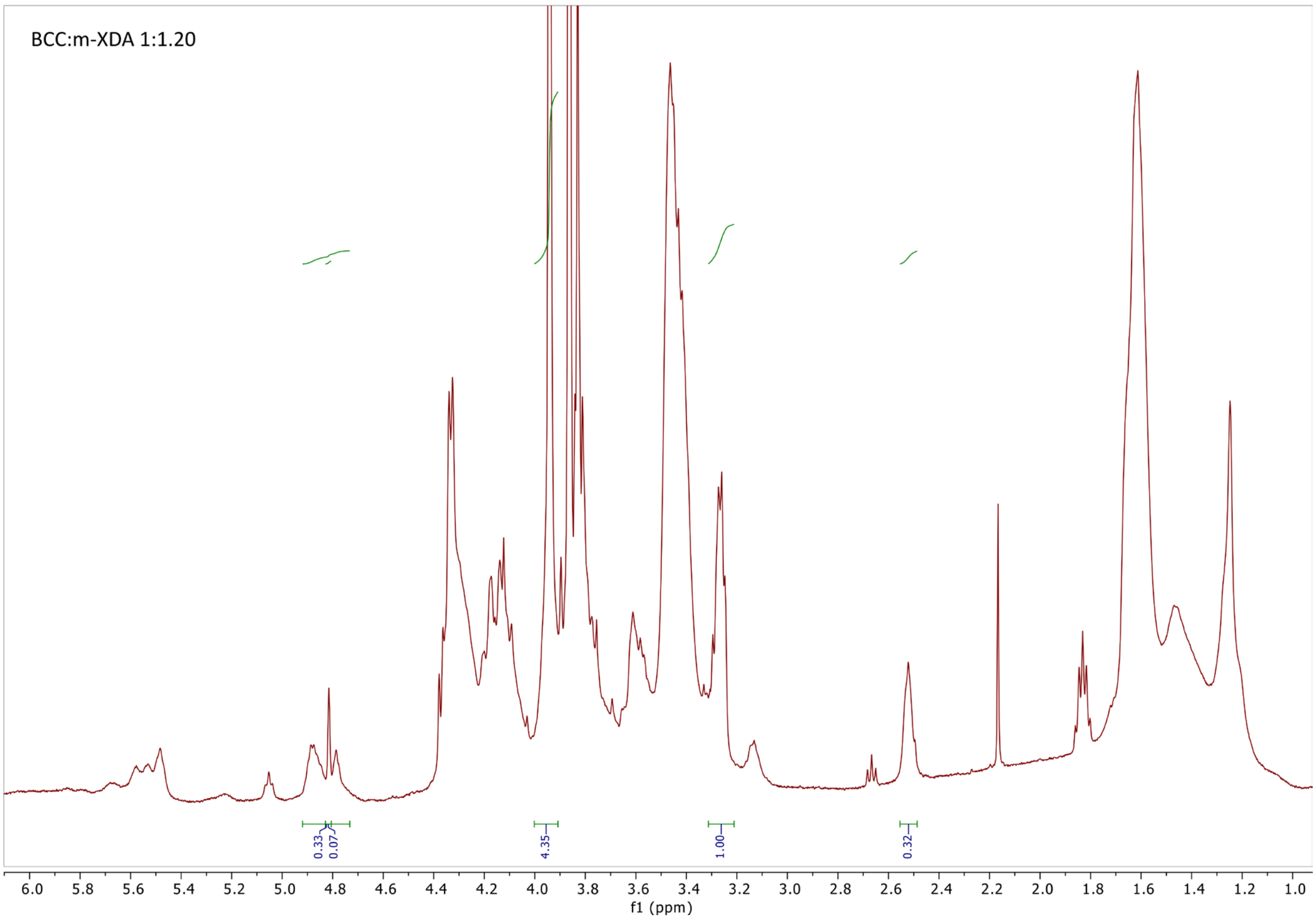

Figure S2. ^{1}H NMR Spectrum of the BCC:m-XDA 1:1.20 PHU pre-polymer in $CDCl_3$.

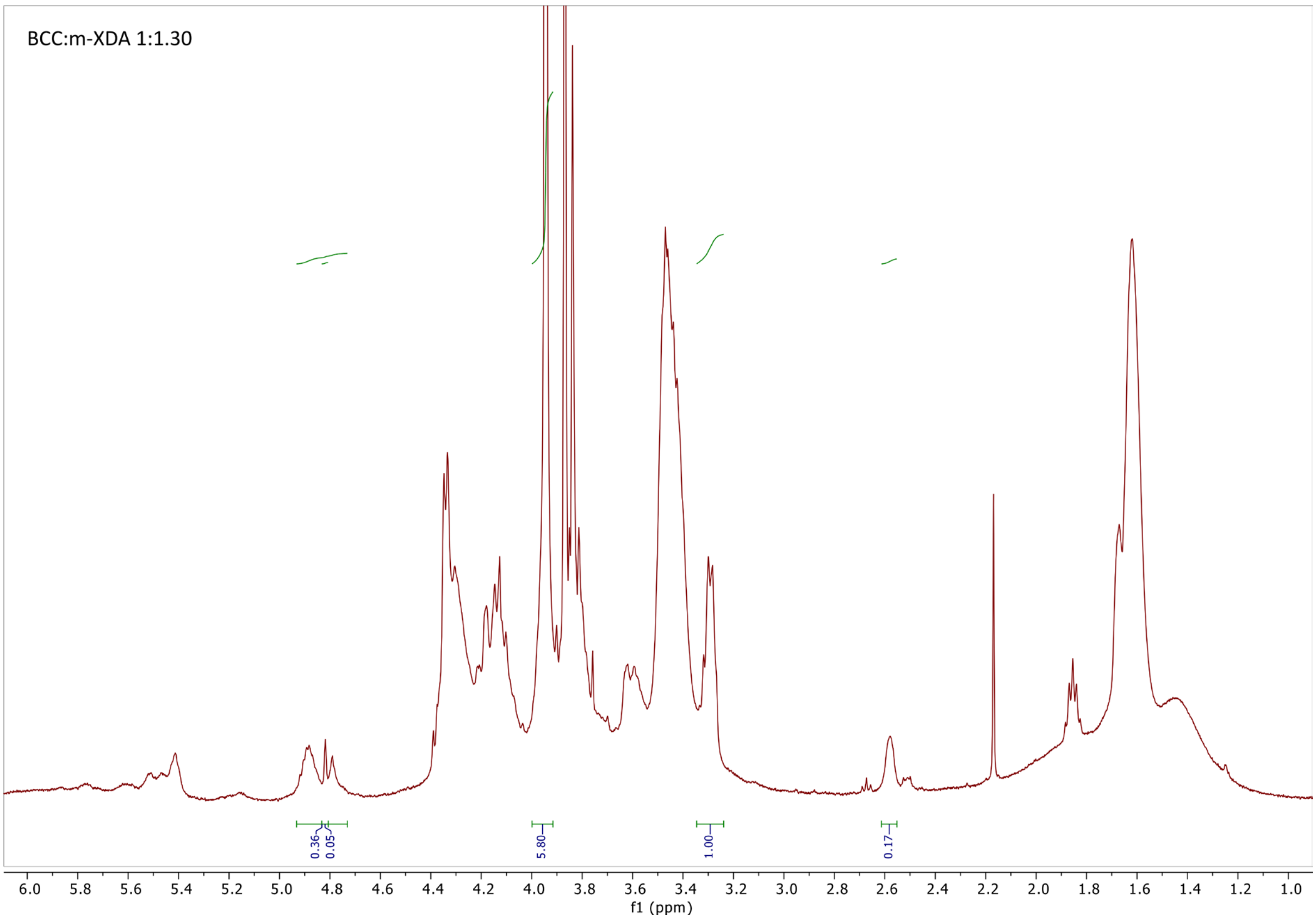

Figure S3. ^{1}H NMR Spectrum of the BCC:m-XDA 1:1.30 PHU pre-polymer in $CDCl_3$.

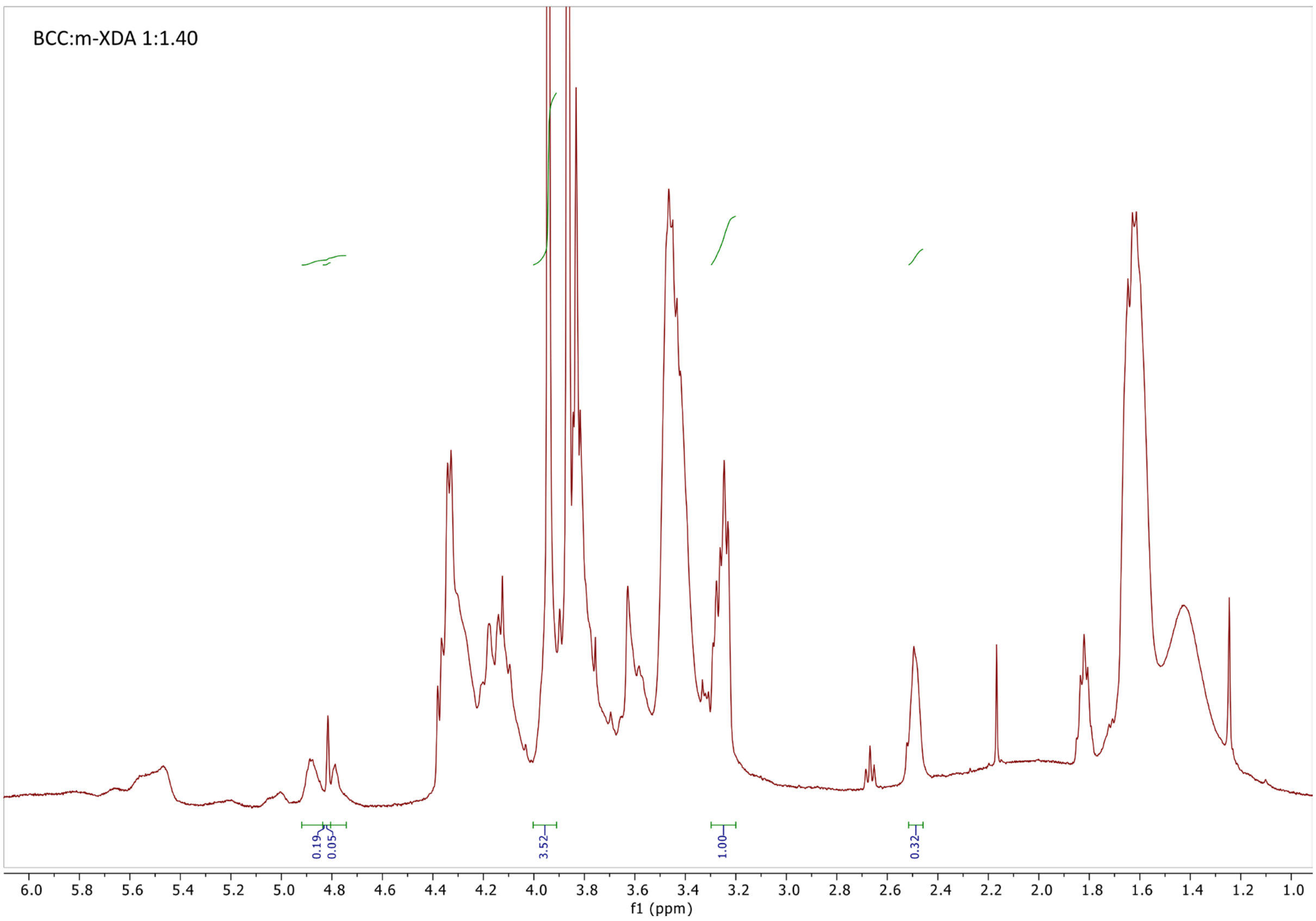

Figure S4. ^{1}H NMR Spectrum of the BCC:m-XDA 1:1.40 PHU pre-polymer in $CDCl_3$.

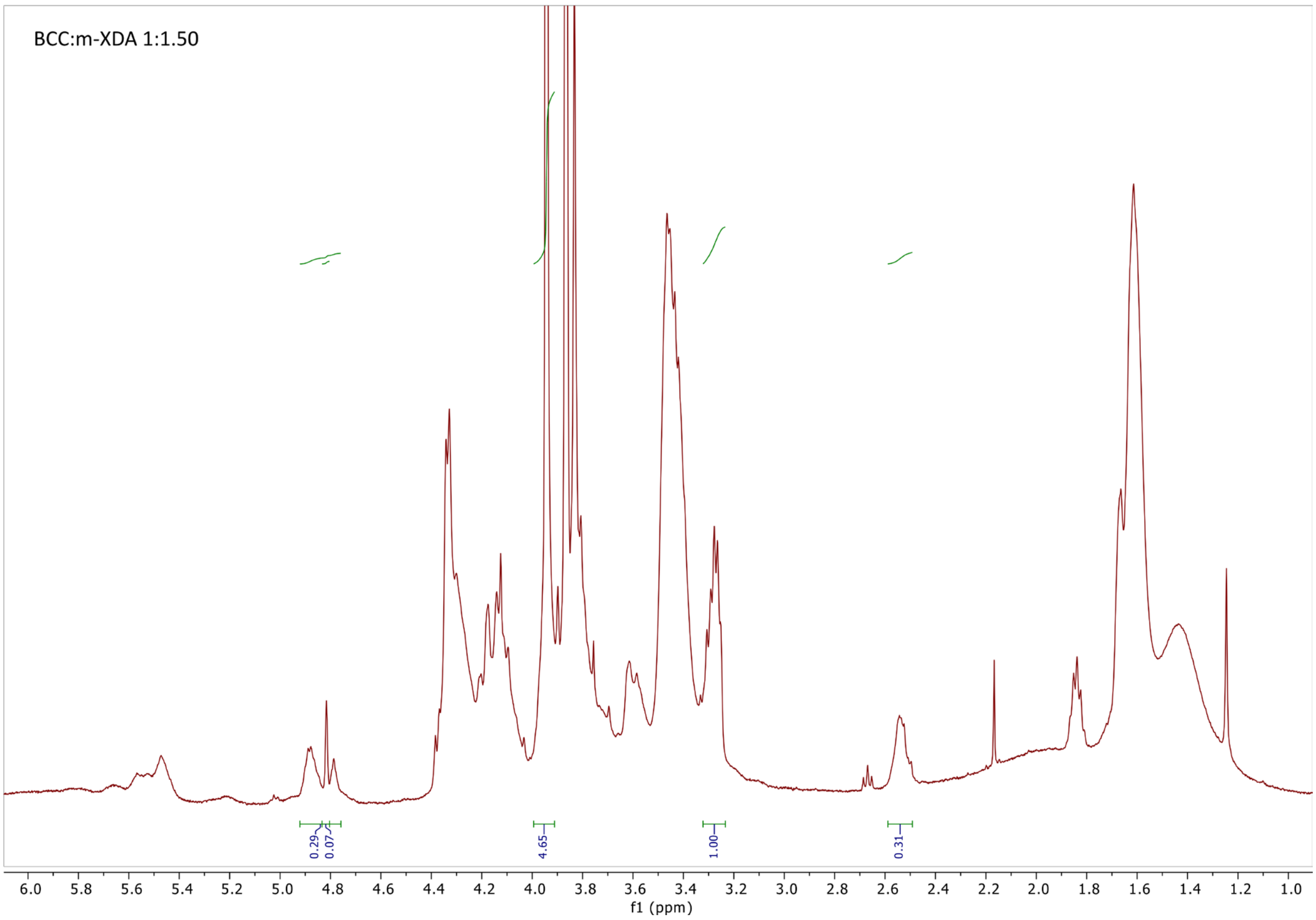

Figure S5. ^{1}H NMR Spectrum of the BCC:m-XDA 1:1.50 PHU pre-polymer in $CDCl_3$.

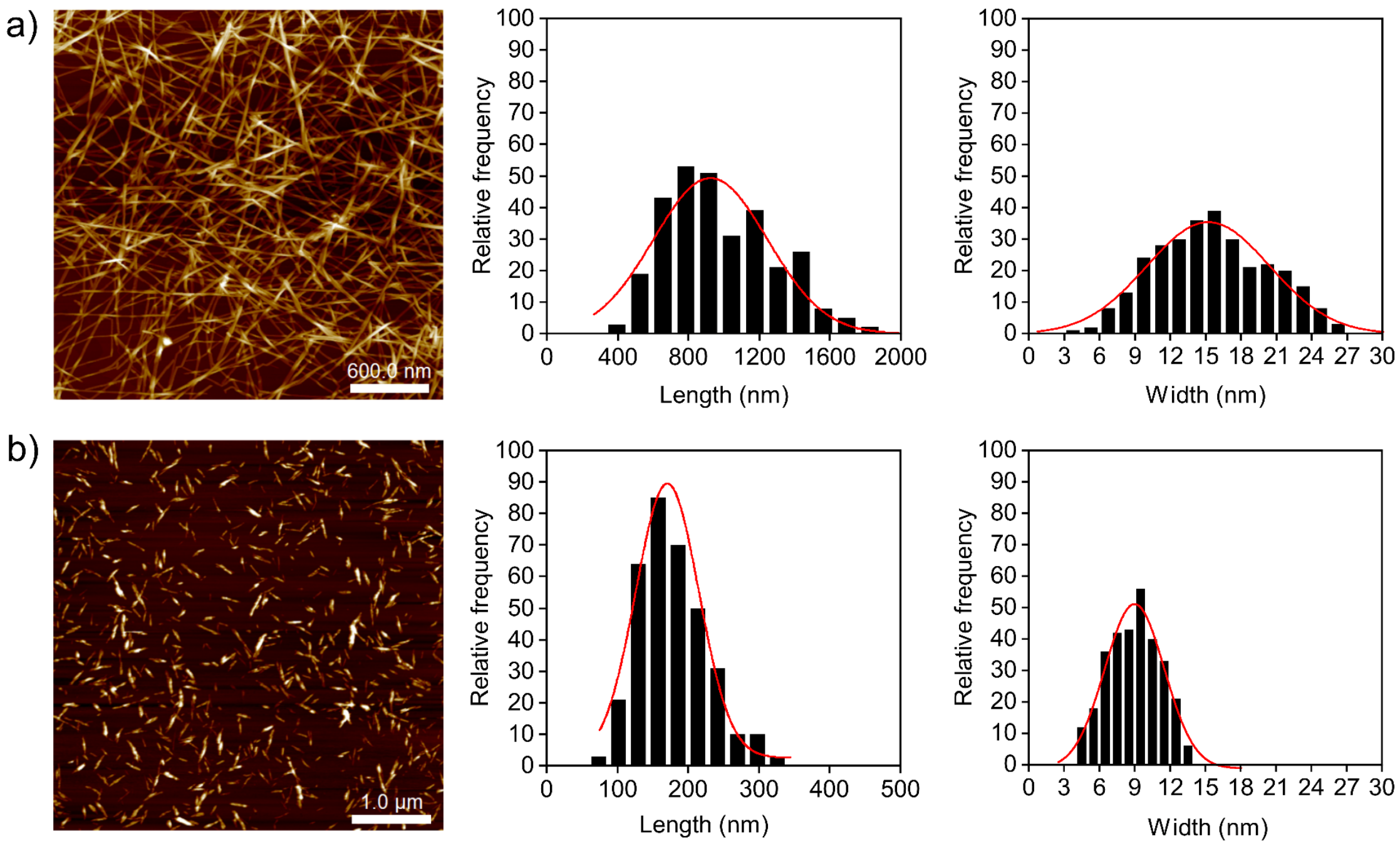

Figure S6. AFM height images for (a) CNC-T and (b) CNC-W, and corresponding histograms showing their width and length distributions.

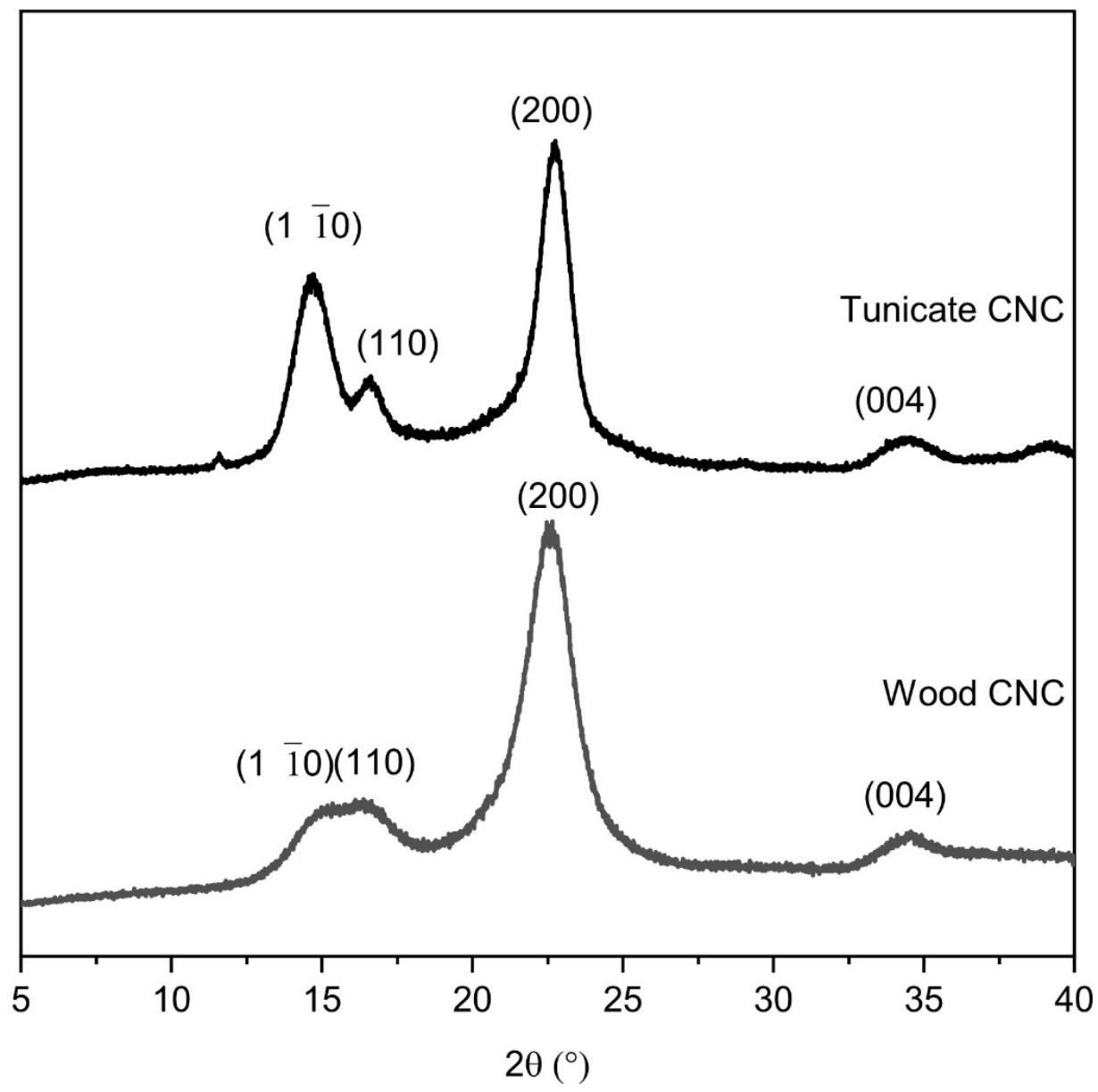

Figure S7. XRD pattern of wood-derived and tunicate-derived CNCs.

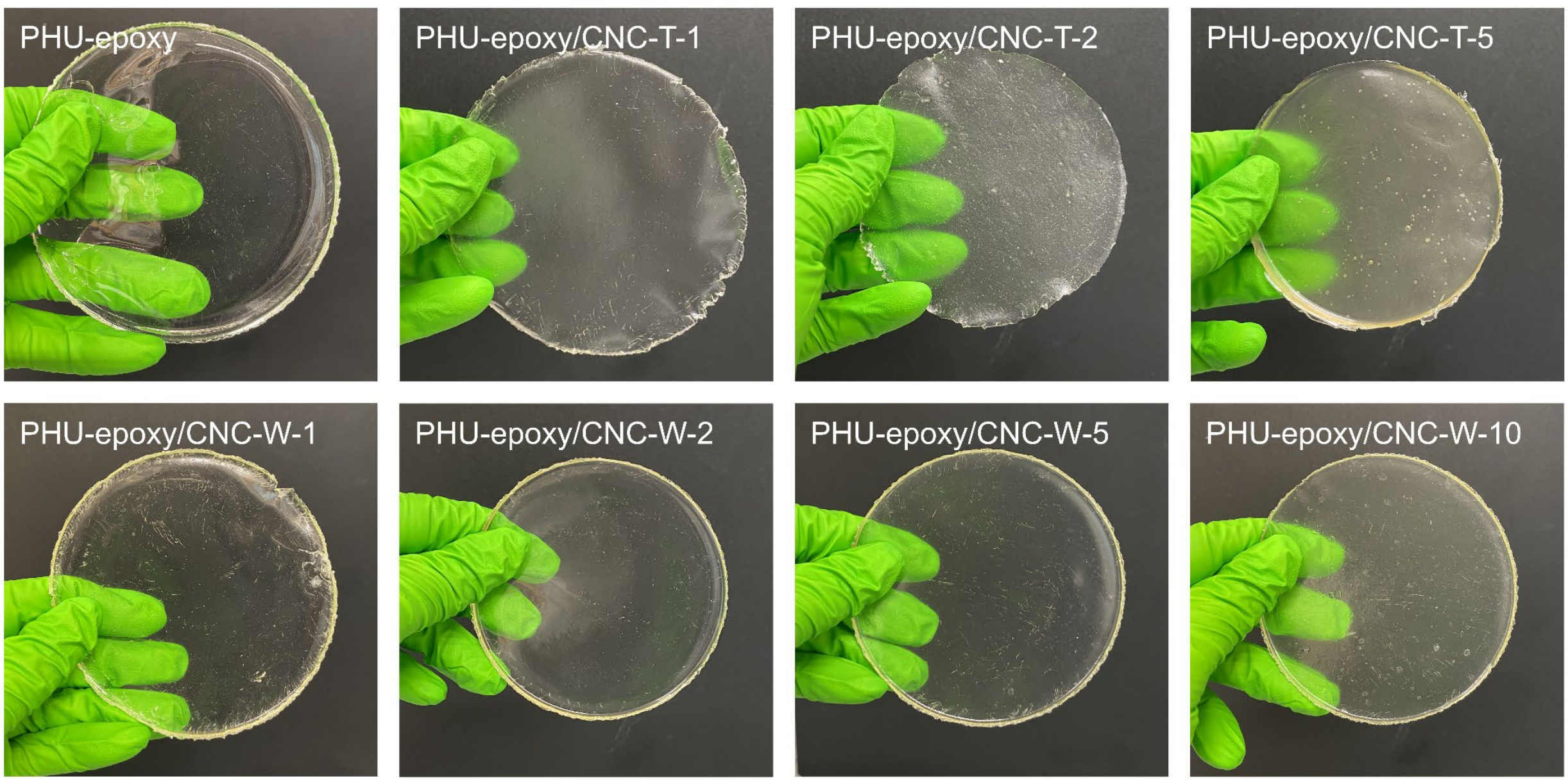

Figure S8. Photographs the PHU–epoxy hybrid polymer and PHU–epoxy/CNC nanocomposites films. The diameter of each film is ~7 cm.

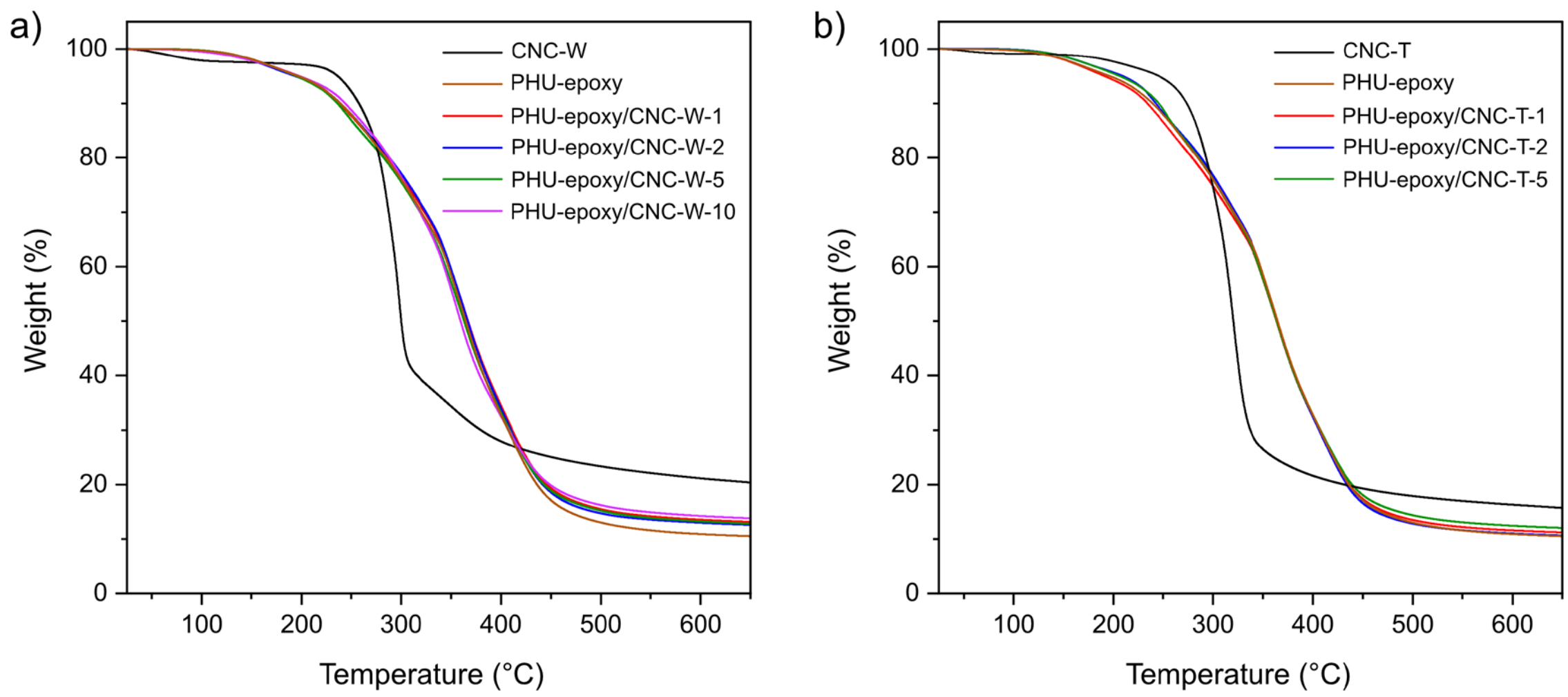

Figure S9. Thermogravimetric analysis (TGA) curves of (a) PHU–epoxy/CNC-W nanocomposites and (b) PHU–epoxy/CNC-T nanocomposites compared with neat PHU–epoxy, CNC-W, and CNC-T.

Table S1. Cyclic carbonate conversion determined by ^{1}H NMR and estimated residual primary amine content in the amine-terminated PHU prepolymers synthesized from different BCC:m-XDA molar ratios. Residual amine content was calculated from the measured cyclic carbonate conversion assuming a 1:1 reaction between cyclic carbonate and amine groups.

BCC : m-XDA	CC conversion (%)	Estimated residual NH_2 (equiv.)
1:1.10	93%	0.17
1:1.20	93%	0.27
1:1.30	94%	0.36
1:1.40	95%	0.45
1:1.50	94%	0.56
